# Integration of an Intensive Care Unit Visualization Dashboard (i-Dashboard) as a Platform to Facilitate Multidisciplinary Rounds: Cluster-Randomized Controlled Trial

**DOI:** 10.2196/35981

**Published:** 2022-05-13

**Authors:** Chao-Han Lai, Kai-Wen Li, Fang-Wen Hu, Pei-Fang Su, I-Lin Hsu, Min‑Hsin Huang, Yen‑Ta Huang, Ping-Yen Liu, Meng-Ru Shen

**Affiliations:** 1 Department of Surgery National Cheng Kung University Hospital, College of Medicine National Cheng Kung University Tainan City Taiwan; 2 Department of Biochemistry and Molecular Biology College of Medicine National Cheng Kung University Tainan City Taiwan; 3 Department of Biostatistics Vanderbilt University Medical Center Nashville, TN United States; 4 Department of Nursing National Cheng Kung University Hospital, College of Medicine National Cheng Kung University Tainan City Taiwan; 5 Department of Statistics College of Management National Cheng Kung University Tainan City Taiwan; 6 Division of Cardiology, Department of Internal Medicine National Cheng Kung University Hospital, College of Medicine National Cheng Kung University Tainan City Taiwan; 7 Institute of Clinical Medicine College of Medicine National Cheng Kung University Tainan City Taiwan; 8 Department of Clinical Medical Research National Cheng Kung University Hospital, College of Medicine National Cheng Kung University Tainan City Taiwan; 9 Department of Obstetrics and Gynecology National Cheng Kung University Hospital, College of Medicine National Cheng Kung University Tainan Taiwan; 10 Department of Pharmacology College of Medicine National Cheng Kung University Tainan City Taiwan

**Keywords:** Intensive care unit, multidisciplinary round, visualization dashboard, large screen, information management strategy, electronic health record, medical record, digital health, dashboard, i-Dashboard, electronic medical record, information exchange

## Abstract

**Background:**

Multidisciplinary rounds (MDRs) are scheduled, patient-focused communication mechanisms among multidisciplinary providers in the intensive care unit (ICU).

**Objective:**

*i*-Dashboard is a custom-developed visualization dashboard that supports (1) key information retrieval and reorganization, (2) time-series data, and (3) display on large touch screens during MDRs. This study aimed to evaluate the performance, including the efficiency of prerounding data gathering, communication accuracy, and information exchange, and clinical satisfaction of integrating *i*-Dashboard as a platform to facilitate MDRs.

**Methods:**

A cluster-randomized controlled trial was performed in 2 surgical ICUs at a university hospital. Study participants included all multidisciplinary care team members. The performance and clinical satisfaction of *i*-Dashboard during MDRs were compared with those of the established electronic medical record (EMR) through direct observation and questionnaire surveys.

**Results:**

Between April 26 and July 18, 2021, a total of 78 and 91 MDRs were performed with the established EMR and *i*-Dashboard, respectively. For prerounding data gathering, the median time was 10.4 (IQR 9.1-11.8) and 4.6 (IQR 3.5-5.8) minutes using the established EMR and *i*-Dashboard (*P*<.001), respectively. During MDRs, data misrepresentations were significantly less frequent with *i*-Dashboard (median 0, IQR 0-0) than with the established EMR (4, IQR 3-5; *P*<.001). Further, effective recommendations were significantly more frequent with *i*-Dashboard than with the established EMR (*P*<.001). The questionnaire results revealed that participants favored using *i*-Dashboard in association with the enhancement of care plan development and team participation during MDRs.

**Conclusions:**

*i*-Dashboard increases efficiency in data gathering. Displaying *i*-Dashboard on large touch screens in MDRs may enhance communication accuracy, information exchange, and clinical satisfaction. The design concepts of *i*-Dashboard may help develop visualization dashboards that are more applicable for ICU MDRs.

**Trial Registration:**

ClinicalTrials.gov NCT04845698; https://clinicaltrials.gov/ct2/show/NCT04845698

## Introduction

Medical care in intensive care units (ICUs) consumes a substantial part of the income of many countries worldwide, and the enormous burden continues to grow [1,2]. Integrated multidisciplinary teamwork, a patient-centered model of care in which intensivists and other members from relevant disciplines provide critical care as a team, effectively complements intensivist care and improves outcomes for critically ill medical and surgical patients [3,4]. Multidisciplinary rounds (MDRs; also called *interprofessional rounds*) are mechanisms that involve scheduled discussion among multidisciplinary providers, including physicians, registered nurses, nurse practitioners (NPs), respiratory therapists (RTs), pharmacists, and dietitians, to review clinical information, exchange opinions, and develop a plan of care [5]. Because effective communication among providers is essential to high-quality patient care, failures during this process may potentially impact the safety and outcomes of ICU patients [5-7].

Understanding causes that potentially impede interdisciplinary communication during MDRs may facilitate improvement in the communication quality among multidisciplinary providers. Based on a systematic review of evidence-informed practices for ICU MDRs, poor retrieval of patient information has been identified as a barrier that hinders information exchange [5]. Currently, clinicians manually access patient information from disparate modules in information systems, and data aggregated into electronic medical record (EMR)-generated printouts or handwritten notes are verbalized in MDRs [8,9]. A recent study revealed that nearly 40% of verbalized laboratory data are inaccurately communicated during MDRs, and only 7.8% of data misrepresentations that precipitate erroneous clinical decisions can be detected [8].

One of the objectives of the technological advancements applied to critical care is simplifying all the avenues of information [10]. It appears that visualization dashboards (also called EMR viewers) have great potential to be the solution as these dashboards are known for the efficiency of clinical information management [9,11-13]. Notably, compared to the standard EMR environment, introducing visualization dashboards may not improve perceived satisfaction with MDRs, such as information presentation or team participation and communication [12]. A possible problem is that displaying dashboards on small monitors positioned on a trolley or bedside computers may give unequal access to data and cause a body orientation shift of providers from other participants to monitors [14], thus potentially hampering interdisciplinary communication during MDRs. In addition, because of unequal EMR access for real-time data viewing to recognize errors and the inability to simultaneously listen, process, and verify data, the multidisciplinary care team relies disproportionately on the intensivist to detect data misrepresentations that potentially lead to medical errors [8]. In the era of rapid development of information technology, an integrated information management strategy to facilitate information retrieval and enhance interdisciplinary communication in MDRs remains to be explored.

In this study, we aimed to develop a user experience–oriented platform as an integrated solution to assist MDRs. The *i*-Dashboard is a care team–designed, patient-centered visualization dashboard in which information extracted from different sources was reorganized on the basis of the requirement of different disciplines or transformed into time-series data as needed. During MDRs, *i*-Dashboard is displayed on wall-mounted large touch screens to bring effective visualization to the multidisciplinary care team. We assumed that *i*-Dashboard might aid prerounding data gathering, and integrating *i*-Dashboard displayed on large touchscreens during MDRs might enhance interdisciplinary communication. Thus, the efficiency, communication accuracy, information exchange, and clinical satisfaction of integrating *i*-Dashboard as a platform to facilitate MDRs were evaluated.

## Methods

### Design and Participants

The study was conducted in a 1300-bed university hospital that offers first-line and tertiary referral services for a population of approximately 1.8 million individuals in southern Taiwan. A cluster-randomized controlled trial was conducted in 2 of the 4 surgical ICUs with 10 and 8 beds. The established EMR (control) and *i*-Dashboard (intervention) were randomly assigned as tools to facilitate prerounding information collection and MDRs in the 2 units and exchanged at 2-week intervals.

Before this trial, MDRs have been carried out in the study units for ~5 years. An integrated multidisciplinary care team is composed of at least 1 intensivist, a registered nurse, an NP, an RT, a pharmacist, and a dietitian. All these providers attend MDRs held on a regular schedule 3 times a week. MDRs are conducted only for patients who stay for more than 7 days because patients receiving surgical ICU care for more than 7 days have a high rate of in-hospital mortality [15], and ~75% of patients have a length of stay for 7 days or less in study units.

### Ethical Considerations

This study was registered with ClinicalTrials.gov (NCT04845698). The study protocol was approved by the institutional review board of National Cheng Kung University Hospital (B-ER-110-040). Informed consent was obtained from all participants in the study. This study is reported in accordance with the CONSORT-EHEALTH (Consolidated Standards of Reporting Trials of Electronic and Mobile HEalth Applications and onLine TeleHealth) checklist (Multimedia Appendix 1) [16].

### Established EMR

The established EMR environment applied in the ICU includes the Philips IntelliSpace Critical Care & Anesthesia information system (ICCA; Philips) and the Hospital Information System (HIS) developed by the institutional Department of Information Technology and its subsystems, including the Laboratory Information System (LIS) and the Picture Archiving and Communication System (PACS). Philips ICCA is an ICU-specific EMR system that provides information essential for critical care [17]. Patient data are organized by data category (demographics, vital signs, laboratory data, etc) in a series of tabs or window panels. The HIS supports text data key-in and patient order entry.

### Development and Architecture of i-Dashboard

This study evaluated the performance of the first version of *i*-Dashboard. The *i*-Dashboard (Advantech) was custom-developed under the guidance of multidisciplinary professionals, including physicians, registered nurses, NPs, RTs, pharmacists, and dietitians, rather than only physicians because information for MDRs needs may vary on the basis of the clinical role. Every health care provider working in the surgical ICU participated in developing *i*-Dashboard. Different professionals held preparatory meetings of their own. Before *i*-Dashboard was formally implemented for clinical use, the structure and layout were repeatedly revised and tailored to achieve broad acceptance among directors of multidisciplinary providers in the ICU.

The architecture of *i*-Dashboard is summarized in Multimedia Appendix 2. In the static mode, the *i*-Dashboard was designed to substitute as a station whiteboard with lists of patients, information to aid emergency evacuation, and on-duty physicians, nurses, and NPs. Patient-level data in *i*-Dashboard were modified from the MDR checklist and digitally transformed. Therefore, data were preidentified and retrieved from different origins in the established EMR environment, especially ICCA. Instead of database-centered displays, these data were reorganized on the basis of the requirements of different professionals or disciplines to form dashboard pages (ie, an overview page and an RT-pharmacist-dietitian page) and element blocks. In addition to colored signaling for values outside the reference ranges, *i*-Dashboard was designed to support built-in automated calculation of severity scores (eg, Simplified Acute Physiology Score II and Sequential Organ Failure Assessment) and visualization of time-series data to expedite navigation of patient condition. Time-series data (eg, vital signs, laboratory data, or severity scores) that were transformed into line charts can be accessed through the hyperlinks located at the left upper corner of element blocks on the overview page. The pages of time-series data were designed to support both fixed (eg, last 24 hours or last 3 days) and relative custom time frames are available.

**Figure 1:**
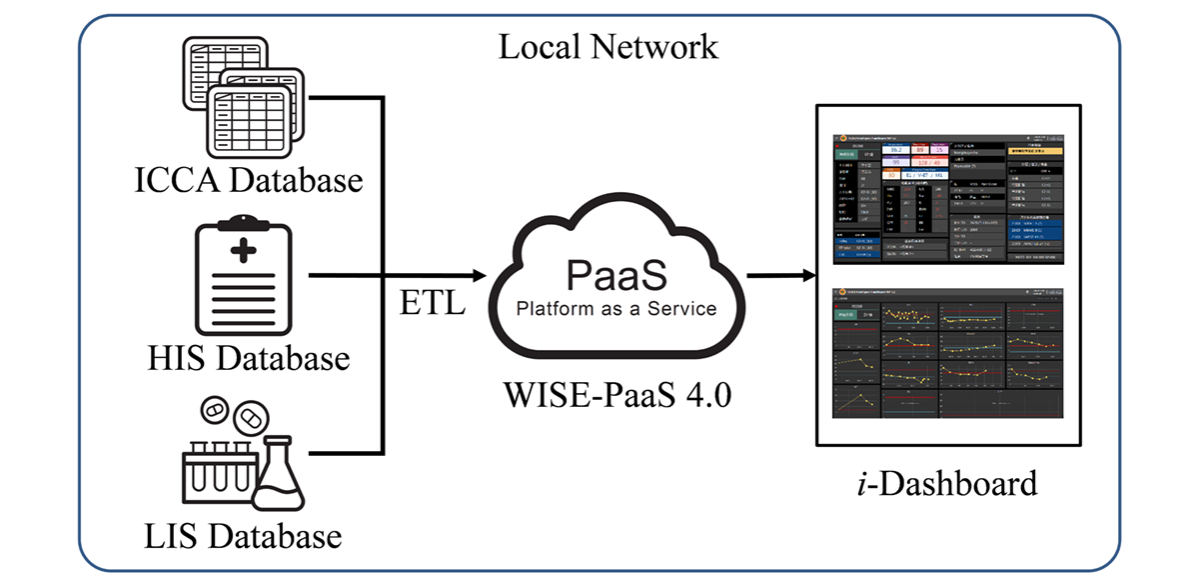
Transfer of the IntelliSpace Critical Care & Anesthesia information system (ICCA), Hospital Information System (HIS), and Laboratory Information System (LIS) data to i-Dashboard. ETL: Extract-Transform-Load. PaaS: Platform as a Service. WISE-PaaS 4.0: brand name of the platform belonging to Advantech.

The technical details underlying *i*-Dashboard are summarized in Figure 1. We used a Windows 10 PC as the visualization platform to support *i*-dashboard. A K8s-based WISE-PaaS 4.0 platform (Advantech) facilitates the integration of diverse devices and communication protocols, making data exchange and system development agile. The entire platform was developed and deployed on 6 VMware servers, each with a 256-GB hard drive and 32-GB memory, and a 24-core Intel Xeon Gold 6248R 3.00 GHz processor. The servers received the data from the Philips ICCA, HIS, and LIS database servers.

In the study units, both the established EMR and *i*-Dashboard can be accessed through desktop computers with 17- or 22-inch monitors or mobile platforms.

### Prerounding Preparation and MDR With the Established EMR

Prerounding data gathering and MDRs have long been standardized with a structured script for reporting (Multimedia Appendix 3). Without *i*-Dashboard, NPs accessed the established EMR systems, including ICCA, HIS, LIS, and PACS, for data gathering through desktop computers.

MDRs took place outside the patient rooms. To facilitate situation awareness of other participants, NPs delivered the oral case presentation and data communication based on the structured script, including basic information, catheter placement and their duration, vital signs, laboratory data, medications, input or output and nutrition, critical values, major image findings, consultations, and other major events (Multimedia Appendix 3). The intensivist summarized active problems, solicited feedback from nurses, RTs, pharmacists, and dietitians, and, if needed, provided in-depth knowledge on the pathophysiology of the current patient condition. The goals of care were documented.

### Prerounding Preparation and MDRs With i-Dashboard

To ensure effective implementation of *i*-Dashboard, we prepared education materials (Multimedia Appendix 2) in the form of brief presentations for all the health care providers working in study units. Subsequently, the use of *i*-Dashboard was tested consistently for 4 weeks.

**Figure 2:**
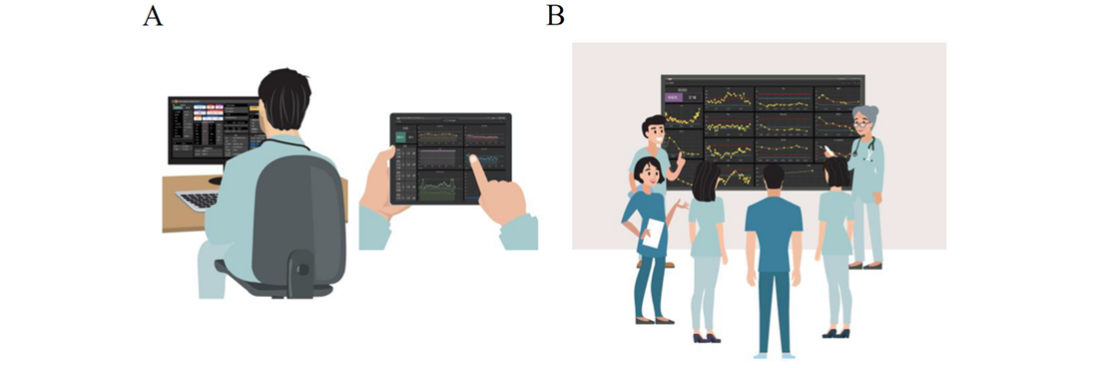
i-Dashboard as a platform to facilitate multidisciplinary rounds. (A) Data access through i-Dashboard on different devices (eg, desktop computers and mobile platforms). (B) i-Dashboard displayed on wall-mounted large touch screens as a visualization aid during multidisciplinary rounds.

As shown in Figure 2, *i*-Dashboard serves as a platform to facilitate MDRs. NPs accessed *i*-Dashboard for data gathering through desktop computers. Instead of ~20 geographically fragmented windows and panels in our established EMR systems, at-a-glance presentations of highly relevant information were displayed on *i*-Dashboard.

During MDRs, all MDR participants gathered in front of a 55-inch 4K interactive touch screen, approximately 3 m away with clear sightlines. The touch screen allowed users to enter different pages of *i*-Dashboard using the finger to tap hyperlinks. The *i*-Dashboard served as a visualization aid for exchanging information and opinions. With the overview page in *i*-Dashboard, the NP carried out patient presentation and data communication. The NP accessed time-series data (eg, laboratory data) through the hyperlinks on the overview page. Time-series data can be rearranged on the basis of different time frames as requested. In addition, the RT, pharmacist, and dietitian could take turns operating *i*-Dashboard and use the RT-pharmacist-dietitian page to demonstrate valuable information of their professionals. Finally, the intensivist used *i*-Dashboard to facilitate bedside teaching. The goals were documented after consensus was reached.

### Data Collection

Two NPs who were not directly involved in MDRs and patient care were trained to audit the processes. The 2 NPs have 8 and 6 years of ICU experience, respectively. The 2 observers were temporarily exempted from clinical work during the study period. To ensure adequate training in the study methodology, personnel piloted data collection and evaluation of communication accuracy were performed by the 2 observers and supervised by the senior investigator (CHL) during a 4-week run-in period. The 2 observers performed in-field observation and audio recordings and audited the process together using a standardized form (Multimedia Appendix 4) to reduce the possibility of losing any useful information and to ensure the correct assessment. Before MDRs, observers measured the amount of time for prerounding data gathering by the NP using the built-in stopwatch app on mobile phones. Subsequently, observers arrived before the start of MDRs and refrained from participating in the discussion during MDRs. Clinical characteristics of patients, information on patient disease severity, and therapeutic interventions during MDRs and established care plans were collected through EMR.

### Primary Outcomes

Primary outcomes of interest included time of data gathering before MDRs and communication accuracy during MDRs. The amount of time that NPs spent gathering clinical data before rounding was recorded. Communication accuracy was evaluated on the basis of the items listed on the standardized form (Multimedia Appendix 4) through direct field observation and audio recordings. Spoken information was compared with EMR data captured by screenshots taken prior to patient presentations. Data communication, including laboratory and nonlaboratory data, was considered inaccurate (ie, data misrepresentation) when the values or data were not correctly reported. For laboratory data communication, only abnormal laboratory data points (outside the reference ranges) were assessed. Laboratory misrepresentations were further classified into several categories as previously defined [8], including omission, old data, pending results, misinterpretation, and erroneous values.

### Secondary Outcomes

Secondary outcomes included information exchange, using effective recommendations initiated by RTs, pharmacists, and dietitians as an index, and clinical satisfaction on *i*-Dashboard. The recommendations initiated by RTs, pharmacists, and dietitians, as exemplified in Multimedia Appendix 5, were considered effective on the condition that they were successfully adopted into the care plan documented in the EMR. Clinical satisfaction with *i*-Dashboard as an information management tool was investigated using Likert scale–based questionnaires (Multimedia Appendix 6) from previously validated survey instruments with minor modifications [12]. Questionnaire 1 was designed to capture the perceived efficiency, accuracy, and safety of the established EMR and *i*-Dashboard was implemented when used to prepare for data gathering and to assist MDRs. The responses were grouped into four dimensions: task productivity, task innovation, customer satisfaction, and management control. Questionnaire 2 was designed to identify intention to use and personal impact of *i*-Dashboard as a result of *i*-Dashboard implementation. At the immediate end of the study, the 2 surveys were administered in hard copy form to study participants. Each participant responded only once.

### Sample Size Estimation and Statistical Analysis

Before the trial began, it was estimated that 12 MDRs would be observed per unit in a 2-week period. To estimate the required sample size, we used a cluster-randomized controlled trial design to account for the positive intraclass correlation expected among members of the same group or cluster. Observational pilot data for prerounding data gathering were collected. The results showed that the mean time difference between the use of the established EMR and *i*-Dashboard was 3 (variance 4) minutes. The intraclass correlation coefficient of this pilot study was 0.47. The corresponding estimations were treated as true parameters. Thus, enrolling approximately 144 patients during 24 unit-weeks would provide a power of 90% at a type I error rate of 0.05 to detect an intervention effect of 3 minutes between groups. Categorical variables were analyzed using the chi-square test or Fisher exact test as needed. Continuous variables and the Likert scale were analyzed using the Mann-Whitney *U* test. Statistical analyses were performed using R software (version 3.4.3; The R Foundation). A 2-tailed *P* value of <.05 was considered statistically significant.

## Results

### Participants

**Figure 3:**
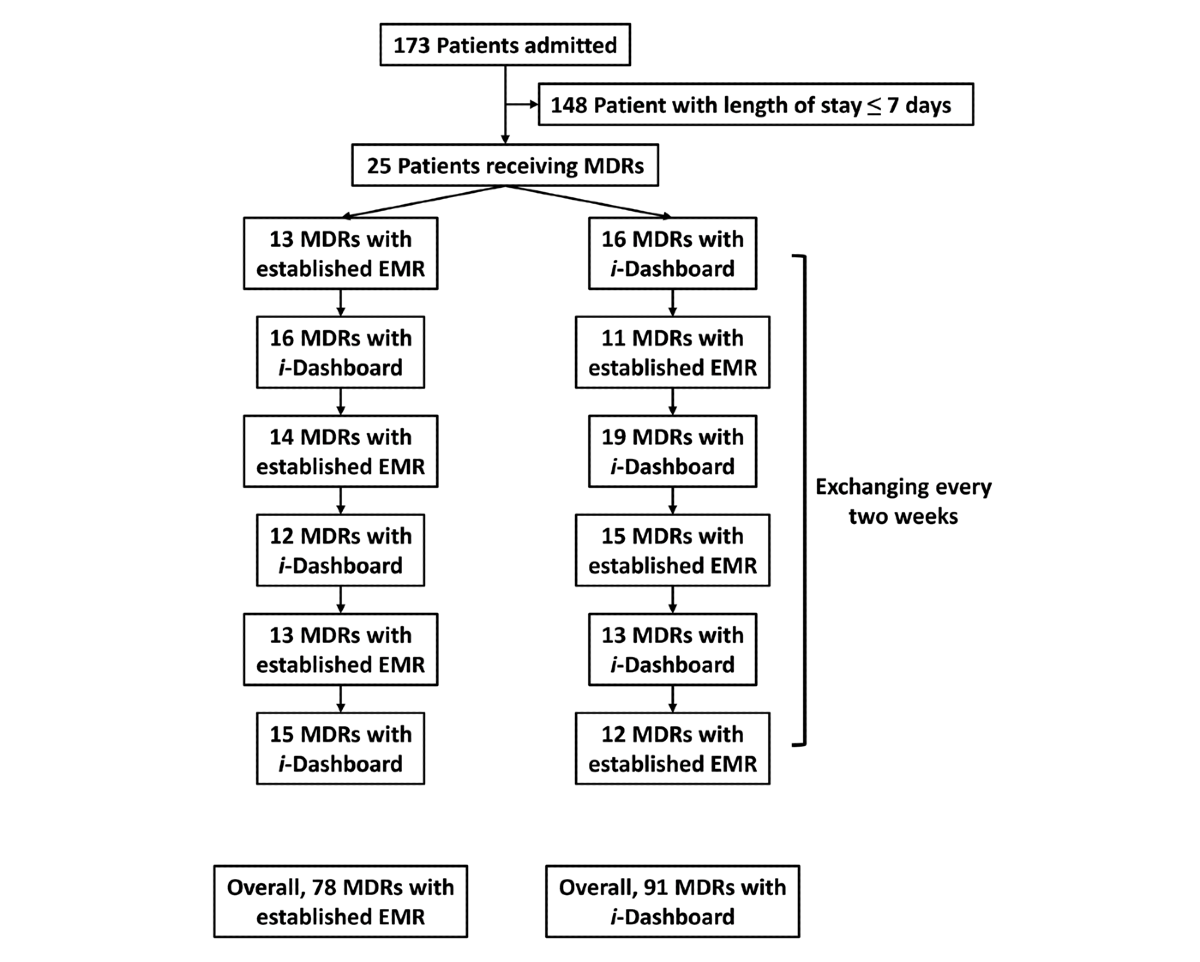
Study flowchart. EMR: electronic medical record, MDR: multidisciplinary round.

Between April 26 and July 18, 2021, there were 173 admissions to the 2 study units. A total of 90 multidisciplinary providers (Table 1) participated in MDRs for the 25 individual patients (Table 2); 78 MDRs were performed with the established EMR environment, whereas 91 MDRs were performed with *i*-Dashboard (Figure 3).

**Table 1:** Characteristics of the 90 multidisciplinary providers.

Variable		Value, n (%)
**Sex**		
	Female	75 (83.3)
	Male	15 (16.7)
**Profession or discipline**		
	Physician	9 (10.0)
	Nurse practitioner	6 (6.7)
	Nurse	51 (56.7)
	Respiratory therapist	20 (22.2)
	Pharmacist	2 (2.2)
	Dietitian	2 (2.2)
**Intensive care unit experience (years)**		
	<1	3 (3.3)
	1-2	20 (22.3)
	3-4	15 (16.7)
	5-9	31 (34.4)
	>10	21 (23.3)

**Table 2:** Clinical characteristics of the 25 patients.

Variable			Value
Age (years), median (IQR)			70 (58-73)
**Age distribution (years), n (%)**			
		<60	7 (28.0)
		60-79	17 (68.0)
		>80	1 (4.0)
**Sex, n (%)**			
	Female		11 (44.0)
	Male		14 (56.0)
**Specialty, n (%)**			
	General surgery		8 (32.0)
	Neurosurgery		11 (44.0)
	Cardiovascular surgery		5 (2.0)
	Trauma surgery		1 (4.0)
**Type of admission, n (%)**			
	Medical		10 (40.0)
	Scheduled surgical		7 (28.0)
	Unscheduled surgical		8 (32.0)
**Acute Physiology and Chronic Health Evaluation II score on admission, n (%)**			
	<15		1 (4.0)
	15-34		21 (84.0)
	>35		3 (12.0)
Mortality, n (%)			2 (8.0)

### Primary Outcomes

Disease severity, in terms of severity scores, and therapeutic interventions at the day of MDRs were not different between the 2 groups (Table 3). The median time for prerounding data gathering was 10.4 (IQR 9.1-11.8) minutes and 4.6 (IQR 3.5-5.8) minutes per patient using the established EMR and *i*-Dashboard (*P*<.001; Table 3), respectively, indicating a reduction of 5.8 (95% CI 5.2-6.4) minutes when using *i*-Dashboard.

Regarding communication accuracy during MDRs (Table 3), data misrepresentations were significantly less frequent in MDRs with *i*-Dashboard (median 0, IQR 0-0) than with the established EMR environment (median 4, IQR 3-5; *P*<.001). In addition, both laboratory and nonlaboratory data misrepresentations were reduced using *i*-Dashboard compared with the established EMR. Among audited laboratory results, only one misrepresentation (0.2%) occurred among 577 data points with *i*-Dashboard. In contrast, 163 (32.3%) misrepresentations occurred in 505 data points with the established EMR environment (*P*<.001), and the majority (95.1%) of these misrepresentations were omissions (155 data points).

**Table 3:** Disease severity and therapeutic intervention at the moment of MDRs and outcomes with the established EMR environment and i-Dashboard.

Variable				Established electronic medical record (n=78)		*i-*Dashboard (n=91)		*P* value
**Severity scoring, median (IQR)**								
	Modified Early Warning Score			6 (5-8)		7 (5-8)		.95
	Simplified Acute Physiology Score II			52 (39-61)		50 (42-62)		.92
	Sequential Organ Failure Assessment			8 (5-11)		7 (4-11)		.57
	Therapeutic Intervention Scoring System-28			35 (32-38)		33 (30-39)		.19
**Therapeutic intervention, n (%)**								
	Mechanical ventilation			72 (92.3)		86 (94.5)		.56
	Vasoactive drug support			18 (23.1)		32 (35.2)		.08
	Mechanical support			3 (3.8)		0 (0)		.10
	Total parenteral nutrition			29 (37.2)		30 (33.0)		.57
	Complicated wound management			24 (30.8)		23 (25.3)		.43
	Dialysis-requiring renal failure			11 (14.1)		13 (14.3)		.73
**Outcome**								
	Time spent on data gathering (minutes), median (IQR)			10.4 (9.1-11.8)		4.6 (3.5-5.8)		<.001
	**Data misrepresentation, median (IQR)**			4 (3-5)		0 (0-0)		<.001
		Laboratory data	2 (1-3)		0 (0-0)		<.001	
		Nonlaboratory data	2 (1-3)		0 (0-0)		<.001	
	**Effective recommendations, n (%)**							<.001
		0	16 (20.5)		15 (16.5)			
		1	41 (52.6)		34 (37.4)			
		2	18 (23.1)		25 (27.5)			
		3	3 (3.8)		17 (18.7)			

### Secondary Outcomes

Regarding information exchange (Table 3), the proportions of 0 and 1 recommendations were lower in MDRs with *i*-Dashboard than with the established EMR, whereas the proportions of 2 and 3 recommendations were higher in MDRs with *i*-Dashboard than with the established EMR. The number of effective recommendations was significantly higher in MDRs with *i*-Dashboard than with the established EMR (*P*<.001).

A total of 76 health care providers (Table S1 in Multimedia Appendix 7) responded to the survey request of clinical satisfaction of *i*-Dashboard (response rate=84.4%). Grouping results of responses to Questionnaire 1 in term of task productivity, task innovation, customer satisfaction, and management control are shown in Table 4, and details are shown in Table S2 in Multimedia Appendix 7. Grouping results revealed that *i*-Dashboard was superior to the established EMR in task productivity (mean 15.91, SD 2.28 vs 14.14, SD 2.35; *P*<.001). Further, *i*-Dashboard was superior to established EMR in task innovation (12.11, SD 1.92 vs 10.41, SD 1.97; *P*<.001), customer satisfaction (16.68, SD 2.02 vs 15.62, SD 2.27; *P*=.002), and management control (16.75, SD 2.21 vs 15.03, SD 2.30; *P*<.001). These findings suggest that *i*-Dashboard outperformed the established EMR across the 4 dimensions.

Finally, survey responses to questionnaire 2 (Table S3 in Multimedia Appendix 7) suggested that these participants were willing to use *i*-Dashboard continuously in association with the enhancement of situation awareness, care plan development, and team participation, and with a reduction in workload and complexity.

**Table 4:** Grouping results of responses to questionnaire 1 in terms of task productivity, task innovation, customer satisfaction, and management control (n=76).

Question		Established electronic medical record, mean (SD)^a^	*i-*Dashboard, mean (SD)^a^	*P* value
**Task productivity**		14.14 (2.35)	15.91 (2.28)	<.001
	Q1. _____ provides information catching up with condition changes.	3.68 (0.79)	4.17 (0.70)	
	Q4. I get the information that I need in time using _____.	3.82 (0.69)	4.12 (0.59)	
	Q5. I get the information that I need using _____ easily.	3.61 (0.80)	4.20 (0.69)	
	Q9. _____ makes data gathering difficult.	3.04 (0.93)	3.42 (1.04)	
**Task innovation**		10.41 (1.97)	12.11 (1.92)	<.001
	Q10. Data gathering with _____ was a mentally demanding task.	2.91 (1.00)	3.58 (1.07)	
	Q14. Communication and opinion exchange in MDRs^b^ is enhanced using _____.	3.75 (0.77)	4.25 (0.66)	
	Q15. Developing care plans relies on joint decisions by team members using _____.	3.75 (0.73)	4.28 (0.65)	
**Customer satisfaction**		15.62 (2.27)	16.68 (2.02)	.002
	Q2. _____ provides information that meets my demand for following MDRs.	4.12 (0.63)	4.29 (0.61)	
	Q3. _____ provides me sufficient information for patient care.	4.00 (0.71)	4.16 (0.63)	
	Q6. I am satisfied with the accuracy of the data using _____.	3.87 (0.68)	4.03 (0.63)	
	Q13. _____ makes me fully understanding the situation and goal of each patient.	3.63 (0.83)	4.21 (0.60)	
**Management control**		15.03 (2.30)	16.75 (2.21)	<.001
	Q7. The information presented by _____ is clear.	3.78 (0.70)	4.25 (0.61)	
	Q8. The information presented in the format of _____ is effective and useful.	3.67 (0.76)	4.29 (0.63)	
	Q11. The information presented using _____ during MDRs was accurate.	3.87 (0.62)	4.07 (0.62)	
	Q12. The presentation of patient information during MDRs using _____ was organized.	3.71 (0.71)	4.15 (0.60)	

^a^Values in Q9 and Q10 were calculated by reverse scoring.

^b^MDR: multidisciplinary round.

## Discussion

### Principal Findings

The *i*-Dashboard was developed as a structured, process-oriented information platform for MDRs, where the efficiency of data retrieval, fidelity of data communication, and satisfaction of interdisciplinary communication are all requisites. Through a comprehensive evaluation of the performance of *i*-Dashboard, we found that under similar disease complexity, *i*-Dashboard may increase efficiency in prerounding data gathering compared to the established EMR. More importantly, displaying *i*-Dashboard on large touch screens in MDRs may enhance communication accuracy, information exchange, and clinical satisfaction.

### Clinical Aspects

Information overload has been a severe problem in the ICU [18]. Critical care providers express frustration with the difficulty in organizing data, especially quantitative dynamic data (eg, deteriorating serum creatinine levels during acute kidney injury), and become overwhelmed by data overload [9,12,13,19]. In time-sensitive care environments, such as the emergency department and the ICU, visualization may provide information that can be readily perceived, easily recognized, and processed expeditiously into inferences [20]. In addition, visualization through dashboards may provide memory aids [11,13,20]. Implementing visualization dashboards in the clinical setting may improve data display and reduce cognitive overload among clinicians [7,9,11,13,21].

This study proposes the notion that large-screen visualization dashboards may improve data communication and information exchange during ICU MDRs. In the emergency department, large-screen visualization dashboards help health care providers find the desired information without wasting time [20,21]. In our study, *i*-Dashboard displayed on large, wall-mounted monitors could present well-organized data visually for the multidisciplinary care team during MDRs and thus avoid data misrepresentations through verbal communication. Compared with small computer monitors, a large screen display effectively prevents unequal access to data and seems more likely to establish consensus. Thus, effective visualization through *i*-Dashboard displayed on large screens contributes to a better perception of information for decision-making by the multidisciplinary care team. When considering the user experience, dashboard visualization may improve the perception and comprehension of patient-level information [20], thereby removing barriers that hinder information exchange. Additionally, the concern regarding the prohibitive cost for large interactive touch screens [14] has been greatly attenuated.

### Comparison With Previous Work

Visualization dashboards can inform decision-making and support behavior change in public health and health care services [22-25]. A recent review of the literature suggests that the strength of evidence on the effect of ICU visualization dashboards remains low [9]. Of the 4 available randomized controlled trials, only Pickering et al [12] found a significant improvement compared to the pre-existing EMR environment. Time spent for prerounding data gathering efforts is practical to evaluate information tools on MDRs [7]. In their study, participants who had access to the AWARE dashboard significantly decreased the data gathering time from 12 to 9 minutes per patient (*P*<.03). In this study, we estimated a reduction of ~60 mouse clicks per patient using *i*-Dashboard versus the established EMR before the trial. For experienced NPs, this improvement can be translated to a 6-minute reduction, compatible with what has been observed (5.8 minutes) during the trial. Health care providers can reduce cognitive fatigue in the data extraction process and pay attention to more productive information.

The ICU is a dynamic environment in which multiple information pathways and personnel interactions facilitate patient care. Specially trained health care professionals in the ICU rely on interdisciplinary communication to make effective clinical decisions. Reliance on a single individual in patient data communication during MDRs represents a coping strategy for an EMR system that does not automatically provide an effective visualization display of the data needed [8]. Consistent with previous studies [8], we found that verbally shared communication of patient data during MDRs was prone to errors and inaccuracies, and most of the laboratory misrepresentations were omissions. Regardless of laboratory or nonlaboratory data, these misrepresentations could be almost eliminated using *i*-Dashboard as a visualization aid for MDRs.

While structured presentation using checklists and explicit definitions of each health care provider’s role may facilitate MDRs, allied health care provider perceptions of not being valued by rounding intensivists have been recognized as an unfavorable factor that impairs the productivity of MDRs. In MDRs with *i*-Dashboard, participants of different professions had a space and time interval of their own, thereby enhancing the sense of participation and collaboration. *i*-Dashboard may help the RT, pharmacist, and dietitian focus on expressing their thoughts explicitly, as other participants could obtain numerical information from the visualization aid. Their inputs were thus more likely to be valued and adopted by the multidisciplinary care team. The positive impacts of *i*-Dashboard on objective outcomes were corroborated by the increased perception of situation awareness and team participation in participants, as revealed in the questionnaire results.

We found that after the study period, health care providers in study units started to propose novel ideas that might improve the usability of *i*-Dashboard (eg, rearranging the layout and increasing laboratory items). A recent study has demonstrated valuable experience regarding the evolution process of an emergency department dashboard, which has undergone several significant revisions to respond to feedback from users [21]. Currently, we are developing the second version of *i*-Dashboard.

### Limitations

Our findings must be interpreted within the context of the study limitations. First, traditional study outcomes in critical care, such as mortality and length of stay, were not evaluated in this study. No conclusion can be achieved regarding the effect of *i*-Dashboard on patient outcomes. Further studies are warranted in this respect. Second, the designers of *i*-Dashboard were part of the team that conducted the study and assessed the outcomes. Successful implementation of dashboards greatly relies on user experience. However, the participation of this paper’s authors in the development process potentially leads to biases in assessing outcomes [26]. Finally, *i*-Dashboard was custom-developed with reference to our established EMR environment. Therefore, it is difficult to extrapolate our study findings directly into ICUs of other hospitals, possibly limiting their generalizability. Nevertheless, visualization dashboards are intended to reduce the time spent on the data gathering process and improve situation awareness and navigation [11,21]. The promising results of *i*-Dashboard obtained in a mature MDR environment suggest that these design concepts may help develop or modify visualization dashboards in the ICU more applicably for MDRs through technological advancements.

### Conclusions

In conclusion, we developed *i*-Dashboard as an information management platform for MDRs. The implementation of *i*-Dashboard can increase efficiency in prerounding data gathering. As a visualization aid, *i*-Dashboard displayed on large screens enhances communication accuracy and information exchange during MDRs. Establishing care team–designed visualization dashboards as an integrated information platform may reinforce the communication quality of MDRs, thus potentially improving the workflow process in the ICU.

## Appendix

Multimedia Appendix 1 CONSORT-EHEALTH checklist.

Multimedia Appendix 2 Architecture of i-Dashboard.

Multimedia Appendix 3 Structured script for data gathering and patient presentation.

Multimedia Appendix 4 Standardized form for evaluating communication accuracy.

Multimedia Appendix 5 Recommendations initiated by respiratory therapists, pharmacists and dietitians.

Multimedia Appendix 6 Questionnaires.

Multimedia Appendix 7 Questionnaire results.

## Abbreviations

EMR: electronic medical record
HIS: Hospital Information Systems
ICCA: IntelliSpace Critical Care & Anesthesia information system
ICU: intensive care unit
LIS: Laboratory Information System
MDR: multidisciplinary round
NP: nurse practitioner
PACS: Picture Archiving and Communication System
RT: respiratory therapist
