# Supplementary material for: Integration of an Intensive Care Unit Visualization Dashboard (i-Dashboard) as a Platform to Facilitate Multidisciplinary Rounds: Cluster-Randomized Controlled Trial
Source: J Med Internet Res. 2022 May 13;24(5):e35981. doi: 10.2196/35981 (PMC9143774; doi:10.2196/35981)
Supplement: Multimedia Appendix 1 [file jmir_v24i5e35981_app1.pdf]

CONSORT-EHEALTH (V 1.6.1) - Submission/Publication Form

The CONSORT-EHEALTH checklist is intended for authors of randomized trials evaluating web-based and Internet-based applications/interventions, including mobile interventions, electronic games (incl multiplayer games), social media, certain telehealth applications, and other interactive and/or networked electronic applications. Some of the items (e.g. all subitems under item 5 - description of the intervention) may also be applicable for other study designs.

The goal of the CONSORT EHEALTH checklist and guideline is to be
a) a guide for reporting for authors of RCTs,
b) to form a basis for appraisal of an ehealth trial (in terms of validity)

CONSORT-EHEALTH items/subitems are MANDATORY reporting items for studies published in the Journal of Medical Internet Research and other journals / scientific societies endorsing the checklist.

Items numbered 1., 2., 3., 4a., 4b etc are original CONSORT or CONSORT-NPT (non-pharmacologic treatment) items.
Items with Roman numerals (i., ii, iii, iv etc.) are CONSORT-EHEALTH extensions/clarifications.

As the CONSORT-EHEALTH checklist is still considered in a formative stage, we would ask that you also RATE ON A SCALE OF 1-5 how important/useful you feel each item is FOR THE PURPOSE OF THE CHECKLIST and reporting guideline (optional).

Mandatory reporting items are marked with a red *.
In the textboxes, either copy & paste the relevant sections from your manuscript into this form - please include any quotes from your manuscript in QUOTATION MARKS,
or answer directly by providing additional information not in the manuscript, or elaborating on why the item was not relevant for this study.

YOUR ANSWERS WILL BE PUBLISHED AS A SUPPLEMENTARY FILE TO YOUR PUBLICATION IN JMIR AND ARE CONSIDERED PART OF YOUR PUBLICATION (IF ACCEPTED).
Please fill in these questions diligently. Information will not be copyedited, so please use proper spelling and grammar, use correct capitalization, and avoid abbreviations.

DO NOT FORGET TO SAVE AS PDF _AND_ CLICK THE SUBMIT BUTTON SO YOUR ANSWERS ARE IN OUR DATABASE !!!

Citation Suggestion (if you append the pdf as Appendix we suggest to cite this paper in the caption):
Eysenbach G, CONSORT-EHEALTH Group
CONSORT-EHEALTH: Improving and Standardizing Evaluation Reports of Web-based and Mobile Health Interventions
J Med Internet Res 2011;13(4):e126
URL: http://www.jmir.org/2011/4/e126/

doi: 10.2196/jmir.1923
PMID: 22209829

chaohan.lai@gmail.com (未分享) 切換帳戶

已儲存草稿

*必填

Your name *

First Last

Chao-Han Lai

Primary Affiliation (short), City, Country *

University of Toronto, Toronto, Canada

Tainan City

Your e-mail address *

abc@gmail.com

d303878@mail.hosp.ncku.edu.tw

Title of your manuscript *

Provide the (draft) title of your manuscript.

Integration of an ICU Visualization Dashboard (i-Dashboard) as a Platform to Facilitate Multidisciplinary Rounds: A Cluster Randomized Controlled Trial

Name of your App/Software/Intervention *

If there is a short and a long/alternate name, write the short name first and add the long name in brackets.

ICU visualization dashboard (i-Dashboard)

Evaluated Version (if any)

e.g. "V1", "Release 2017-03-01", "Version 2.0.27913"

V1, release 2021-03-01

Language(s) *

What language is the intervention/app in? If multiple languages are available, separate by comma (e.g. "English, French")

English, Mandarin Chinese

URL of your Intervention Website or App

e.g. a direct link to the mobile app on app in appstore (itunes, Google Play), or URL of the website. If the intervention is a DVD or hardware, you can also link to an Amazon page.

您的回答

URL of an image/screenshot (optional)

您的回答

Accessibility *

Can an enduser access the intervention presently?

- ○ access is free and open
- ◉ access only for special usergroups, not open
- ○ access is open to everyone, but requires payment/subscription/in-app purchases
- ○ app/intervention no longer accessible
- ○ 其他:

Primary Medical Indication/Disease/Condition *

e.g. "Stress", "Diabetes", or define the target group in brackets after the condition, e.g. "Autism (Parents of children with)", "Alzheimers (Informal Caregivers of)"

Multidisciplinary rounds in the ICU

Primary Outcomes measured in trial *

comma-separated list of primary outcomes reported in the trial

Efficiency of pre-rounding data gathering, com

Secondary/other outcomes

Are there any other outcomes the intervention is expected to affect?

Information exchange, clinical satisfaction

Recommended "Dose" *

What do the instructions for users say on how often the app should be used?

- ◯ Approximately Daily
- ◉ Approximately Weekly
- ◯ Approximately Monthly
- ◯ Approximately Yearly
- ◯ "as needed"
- ◯ 其他:

Approx. Percentage of Users (starters) still using the app as recommended after 3 months *

- ◯ unknown / not evaluated
- ◯ 0-10%
- ◯ 11-20%
- ◯ 21-30%
- ◯ 31-40%
- ◯ 41-50%
- ◯ 51-60%
- ◯ 61-70%
- ◯ 71%-80%
- ◯ 81-90%
- ◉ 91-100%
- ◯ 其他:

Overall, was the app/intervention effective? *

- (selected) yes: all primary outcomes were significantly better in intervention group vs control
- partly: SOME primary outcomes were significantly better in intervention group vs control
- no statistically significant difference between control and intervention
- potentially harmful: control was significantly better than intervention in one or more outcomes
- inconclusive: more research is needed
- 其他:

Article Preparation Status/Stage *

At which stage in your article preparation are you currently (at the time you fill in this form)

- not submitted yet - in early draft status
- not submitted yet - in late draft status, just before submission
- submitted to a journal but not reviewed yet
- (selected) submitted to a journal and after receiving initial reviewer comments
- submitted to a journal and accepted, but not published yet
- published
- 其他:

Journal *

If you already know where you will submit this paper (or if it is already submitted), please provide the journal name (if it is not JMIR, provide the journal name under "other")

- ○ not submitted yet / unclear where I will submit this
- ◉ Journal of Medical Internet Research (JMIR)
- ○ JMIR mHealth and UHealth
- ○ JMIR Serious Games
- ○ JMIR Mental Health
- ○ JMIR Public Health
- ○ JMIR Formative Research
- ○ Other JMIR sister journal
- ○ 其他:

Is this a full powered effectiveness trial or a pilot/feasibility trial? *

- ○ Pilot/feasibility
- ◉ Fully powered

Manuscript tracking number *

If this is a JMIR submission, please provide the manuscript tracking number under "other" (The ms tracking number can be found in the submission acknowledgement email, or when you login as author in JMIR. If the paper is already published in JMIR, then the ms tracking number is the four-digit number at the end of the DOI, to be found at the bottom of each published article in JMIR)

- ○ no ms number (yet) / not (yet) submitted to / published in JMIR
- ◉ 其他: JMIR ms#35981

TITLE AND ABSTRACT

1a) TITLE: Identification as a randomized trial in the title

1a) Does your paper address CONSORT item 1a? *

I.e does the title contain the phrase "Randomized Controlled Trial"? (if not, explain the reason under "other")

- (selected) yes
- ( ) 其他:

1a-i) Identify the mode of delivery in the title

Identify the mode of delivery. Preferably use “web-based” and/or “mobile” and/or “electronic game” in the title. Avoid ambiguous terms like “online”, “virtual”, “interactive”. Use “Internet-based” only if Intervention includes non-web-based Internet components (e.g. email), use “computer-based” or “electronic” only if offline products are used. Use “virtual” only in the context of “virtual reality” (3-D worlds). Use “online” only in the context of “online support groups”. Complement or substitute product names with broader terms for the class of products (such as “mobile” or “smart phone” instead of “iphone”), especially if the application runs on different platforms.

	1	2	3	4	5	
subitem not at all important	( )	( )	( )	( )	(selected)	essential

清除選取的項目

Does your paper address subitem 1a-i? *

Copy and paste relevant sections from manuscript title (include quotes in quotation marks "like this" to indicate direct quotes from your manuscript), or elaborate on this item by providing additional information not in the ms, or briefly explain why the item is not applicable/relevant for your study

A cluster randomized trial

1a-ii) Non-web-based components or important co-interventions in title

Mention non-web-based components or important co-interventions in title, if any (e.g., “with telephone support”).

	1	2	3	4	5	
subitem not at all important	◉	○	○	○	○	essential

清除選取的項目

Does your paper address subitem 1a-ii?

Copy and paste relevant sections from manuscript title (include quotes in quotation marks "like this" to indicate direct quotes from your manuscript), or elaborate on this item by providing additional information not in the ms, or briefly explain why the item is not applicable/relevant for your study

Not applicable in the present study

1a-iii) Primary condition or target group in the title

Mention primary condition or target group in the title, if any (e.g., “for children with Type I Diabetes”)
Example: A Web-based and Mobile Intervention with Telephone Support for Children with Type I Diabetes: Randomized Controlled Trial

	1	2	3	4	5	
subitem not at all important	○	○	○	○	◉	essential

清除選取的項目

Does your paper address subitem 1a-iii? *

Copy and paste relevant sections from manuscript title (include quotes in quotation marks "like this" to indicate direct quotes from your manuscript), or elaborate on this item by providing additional information not in the ms, or briefly explain why the item is not applicable/relevant for your study

ICU visualization dashboard (i-Dashboard) as a platform to facilitate multidisciplinary rounds

1b) ABSTRACT: Structured summary of trial design, methods, results, and conclusions

NPT extension: Description of experimental treatment, comparator, care providers, centers, and blinding status.

1b-i) Key features/functionalities/components of the intervention and comparator in the METHODS section of the ABSTRACT

Mention key features/functionalities/components of the intervention and comparator in the abstract. If possible, also mention theories and principles used for designing the site. Keep in mind the needs of systematic reviewers and indexers by including important synonyms. (Note: Only report in the abstract what the main paper is reporting. If this information is missing from the main body of text, consider adding it)

	1	2	3	4	5	
subitem not at all important	○	○	○	○	◉	essential

清除選取的項目

Does your paper address subitem 1b-i? *

Copy and paste relevant sections from the manuscript abstract (include quotes in quotation marks "like this" to indicate direct quotes from your manuscript), or elaborate on this item by providing additional information not in the ms, or briefly explain why the item is not applicable/relevant for your study

i-Dashboard is a custom-developed visualization dashboard that supports 1) key information retrieval and reorganization, 2) time-series data and 3) display on large touchscreens during MDRs.

1b-ii) Level of human involvement in the METHODS section of the ABSTRACT

Clarify the level of human involvement in the abstract, e.g., use phrases like “fully automated” vs. “therapist/nurse/care provider/physician-assisted” (mention number and expertise of providers involved, if any). (Note: Only report in the abstract what the main paper is reporting. If this information is missing from the main body of text, consider adding it)

	1	2	3	4	5	
subitem not at all important	○	○	○	○	◉	essential

清除選取的項目

Does your paper address subitem 1b-ii?

Copy and paste relevant sections from the manuscript abstract (include quotes in quotation marks "like this" to indicate direct quotes from your manuscript), or elaborate on this item by providing additional information not in the ms, or briefly explain why the item is not applicable/relevant for your study

Study participants included all multidisciplinary care team members.

1b-iii) Open vs. closed, web-based (self-assessment) vs. face-to-face assessments in the METHODS section of the ABSTRACT

Mention how participants were recruited (online vs. offline), e.g., from an open access website or from a clinic or a closed online user group (closed usergroup trial), and clarify if this was a purely web-based trial, or there were face-to-face components (as part of the intervention or for assessment). Clearly say if outcomes were self-assessed through questionnaires (as common in web-based trials). Note: In traditional offline trials, an open trial (open-label trial) is a type of clinical trial in which both the researchers and participants know which treatment is being administered. To avoid confusion, use “blinded” or “unblinded” to indicated the level of blinding instead of “open”, as “open” in web-based trials usually refers to “open access” (i.e. participants can self-enrol). (Note: Only report in the abstract what the main paper is reporting. If this information is missing from the main body of text, consider adding it)

	1	2	3	4	5	
subitem not at all important	○	○	○	○	◉	essential

清除選取的項目

Does your paper address subitem 1b-iii?

Copy and paste relevant sections from the manuscript abstract (include quotes in quotation marks "like this" to indicate direct quotes from your manuscript), or elaborate on this item by providing additional information not in the ms, or briefly explain why the item is not applicable/relevant for your study

The performances and clinical satisfaction of i-Dashboard during MDRs were compared with those of the established electronic medical record (EMR) through direct observation and questionnaire survey.

1b-iv) RESULTS section in abstract must contain use data

Report number of participants enrolled/assessed in each group, the use/uptake of the intervention (e.g., attrition/adherence metrics, use over time, number of logins etc.), in addition to primary/secondary outcomes. (Note: Only report in the abstract what the main paper is reporting. If this information is missing from the main body of text, consider adding it)

	1	2	3	4	5	
subitem not at all important	○	○	○	○	◉	essential

清除選取的項目

Does your paper address subitem 1b-iv?

Copy and paste relevant sections from the manuscript abstract (include quotes in quotation marks "like this" to indicate direct quotes from your manuscript), or elaborate on this item by providing additional information not in the ms, or briefly explain why the item is not applicable/relevant for your study

Between April 26, 2021, and July 18, 2021, 78 and 91 MDRs were performed with the established EMR and i-Dashboard, respectively.

1b-v) CONCLUSIONS/DISCUSSION in abstract for negative trials

Conclusions/Discussions in abstract for negative trials: Discuss the primary outcome - if the trial is negative (primary outcome not changed), and the intervention was not used, discuss whether negative results are attributable to lack of uptake and discuss reasons. (Note: Only report in the abstract what the main paper is reporting. If this information is missing from the main body of text, consider adding it)

	1	2	3	4	5	
subitem not at all important	○	○	○	○	◉	essential

清除選取的項目

Does your paper address subitem 1b-v?

Copy and paste relevant sections from the manuscript abstract (include quotes in quotation marks "like this" to indicate direct quotes from your manuscript), or elaborate on this item by providing additional information not in the ms, or briefly explain why the item is not applicable/relevant for your study

Not applicable in the present study (with positive results)

INTRODUCTION

2a) In INTRODUCTION: Scientific background and explanation of rationale

2a-i) Problem and the type of system/solution

Describe the problem and the type of system/solution that is object of the study: intended as stand-alone intervention vs. incorporated in broader health care program? Intended for a particular patient population? Goals of the intervention, e.g., being more cost-effective to other interventions, replace or complement other solutions? (Note: Details about the intervention are provided in “Methods” under 5)

	1	2	3	4	5	
subitem not at all important	○	○	○	○	◉	essential

清除選取的項目

Does your paper address subitem 2a-i? *

Copy and paste relevant sections from the manuscript (include quotes in quotation marks "like this" to indicate direct quotes from your manuscript), or elaborate on this item by providing additional information not in the ms, or briefly explain why the item is not applicable/relevant for your study

Understanding causes that potentially impede interdisciplinary communication during MDRs may facilitate improving the quality of communication among multidisciplinary providers. Based on a systematic review of evidence-informed practices for ICU MDRs, poor retrieval of patient information has been identified as a barrier that hinders information exchange [5]. Currently, clinicians manually access patient information from disparate modules in information systems, and data aggregated into electronic medical record (EMR)-generated printouts or handwritten notes are verbalized in MDRs [8, 9]. A recent study revealed that nearly 40% of verbalized laboratory data are inaccurately communicated during MDRs, and only 7.8% of data misrepresentations that precipitate erroneous clinical decisions can be detected [8].

2a-ii) Scientific background, rationale: What is known about the (type of) system

Scientific background, rationale: What is known about the (type of) system that is the object of the study (be sure to discuss the use of similar systems for other conditions/diagnoses, if appropiate), motivation for the study, i.e. what are the reasons for and what is the context for this specific study, from which stakeholder viewpoint is the study performed, potential impact of findings [2]. Briefly justify the choice of the comparator.

	1	2	3	4	5	
subitem not at all important	○	○	○	○	◉	essential

清除選取的項目

Does your paper address subitem 2a-ii? *

Copy and paste relevant sections from the manuscript (include quotes in quotation marks "like this" to indicate direct quotes from your manuscript), or elaborate on this item by providing additional information not in the ms, or briefly explain why the item is not applicable/relevant for your study

One of the objectives of the technological advancements applied to critical care is simplifying all the avenues of information [10]. It appears that visualization dashboards (also called EMR viewers) have a great potential to be the solution as these dashboards are known for the efficiency of clinical information management [9, 11-13]. Notably, compared to the standard EMR environment, introducing visualization dashboards may not improve perceived satisfaction with MDRs, such as information presentation or team participation and communication [12]. A possible problem is that displaying dashboards on small monitors positioned on a trolley or bedside computers may give unequal access to data and cause a body orientation shift of providers from other participants to monitors [14], potentially hampering interdisciplinary communication during MDRs. In addition, because of unequal EMR access for real-time data viewing to recognize errors and the inability to simultaneously listen, process and verify data, the multidisciplinary care team relies disproportionately on the intensivist to detect data misrepresentations that potentially lead to medical errors [8]. In the era of rapid development of information technology, an integrated information management strategy to facilitate information retrieval and enhance interdisciplinary communication in MDRs remains to be explored.

2b) In INTRODUCTION: Specific objectives or hypotheses

Does your paper address CONSORT subitem 2b? *

Copy and paste relevant sections from the manuscript (include quotes in quotation marks "like this" to indicate direct quotes from your manuscript), or elaborate on this item by providing additional information not in the ms, or briefly explain why the item is not applicable/relevant for your study

In the study presented here, we aimed to develop a user experience-oriented platform as an integrated solution to assist MDRs. The i-Dashboard is a care team-designed, patient-centered visualization dashboard in which information extracted from different sources was reorganized based on the requirement of different disciplines or transformed into time-series data as needed. During MDRs, i-Dashboard is displayed on wall-mounted large touchscreens to bring effective visualization to the multidisciplinary care team. We assumed that i-Dashboard might aid pre-rounding data gathering, and integrating i-Dashboard displayed on large touchscreens during MDRs might enhance interdisciplinary communication. Thus, the efficiency, communication accuracy, information exchange and clinical satisfaction of integrating i-Dashboard as a platform to facilitate MDRs were evaluated.

METHODS

3a) Description of trial design (such as parallel, factorial) including allocation ratio

Does your paper address CONSORT subitem 3a? *

Copy and paste relevant sections from the manuscript (include quotes in quotation marks "like this" to indicate direct quotes from your manuscript), or elaborate on this item by providing additional information not in the ms, or briefly explain why the item is not applicable/relevant for your study

A cluster randomized trial was conducted in two of the four surgical ICUs with 10 and 8 beds. The established EMR (control) and i-Dashboard (intervention) were randomly assigned as tools to facilitate pre-rounding information collection and MDRs in the two units and exchanged at two-week intervals.

3b) Important changes to methods after trial commencement (such as eligibility criteria), with reasons

Does your paper address CONSORT subitem 3b? *

Copy and paste relevant sections from the manuscript (include quotes in quotation marks "like this" to indicate direct quotes from your manuscript), or elaborate on this item by providing additional information not in the ms, or briefly explain why the item is not applicable/relevant for your study

Not applicable in the present study

3b-i) Bug fixes, Downtimes, Content Changes

Bug fixes, Downtimes, Content Changes: ehealth systems are often dynamic systems. A description of changes to methods therefore also includes important changes made on the intervention or comparator during the trial (e.g., major bug fixes or changes in the functionality or content) (5-iii) and other “unexpected events” that may have influenced study design such as staff changes, system failures/downtimes, etc. [2].

	1	2	3	4	5	
subitem not at all important	○	○	○	○	◉	essential

清除選取的項目

Does your paper address subitem 3b-i?

Copy and paste relevant sections from the manuscript (include quotes in quotation marks "like this" to indicate direct quotes from your manuscript), or elaborate on this item by providing additional information not in the ms, or briefly explain why the item is not applicable/relevant for your study

Not applicable in the present study

4a) Eligibility criteria for participants

Does your paper address CONSORT subitem 4a? *

Copy and paste relevant sections from the manuscript (include quotes in quotation marks "like this" to indicate direct quotes from your manuscript), or elaborate on this item by providing additional information not in the ms, or briefly explain why the item is not applicable/relevant for your study

Before this trial, MDRs have been carried out in the study units for ~5 years. An integrated multidisciplinary care team is comprised of at least an intensivist, a registered nurse, a NP, a RT, a pharmacist and a dietitian. All these providers attend MDRs held on a regular schedule three times a week. MDRs are conducted only for patients who stay for more than 7 days because patients receiving surgical ICU care for more than 7 days have a high rate of in-hospital mortality [15], and ~75% of patients have a length of stay for 7 days or less in study units.

4a-i) Computer / Internet literacy

Computer / Internet literacy is often an implicit “de facto” eligibility criterion - this should be explicitly clarified.

	1	2	3	4	5	
subitem not at all important	○	○	○	○	◉	essential

清除選取的項目

Does your paper address subitem 4a-i?

Copy and paste relevant sections from the manuscript (include quotes in quotation marks "like this" to indicate direct quotes from your manuscript), or elaborate on this item by providing additional information not in the ms, or briefly explain why the item is not applicable/relevant for your study

Not applicable in the present study

4a-ii) Open vs. closed, web-based vs. face-to-face assessments:

Open vs. closed, web-based vs. face-to-face assessments: Mention how participants were recruited (online vs. offline), e.g., from an open access website or from a clinic, and clarify if this was a purely web-based trial, or there were face-to-face components (as part of the intervention or for assessment), i.e., to what degree got the study team to know the participant. In online-only trials, clarify if participants were quasi-anonymous and whether having multiple identities was possible or whether technical or logistical measures (e.g., cookies, email confirmation, phone calls) were used to detect/prevent these.

	1	2	3	4	5	
subitem not at all important	○	○	○	○	◉	essential

清除選取的項目

Does your paper address subitem 4a-ii? *

Copy and paste relevant sections from the manuscript (include quotes in quotation marks "like this" to indicate direct quotes from your manuscript), or elaborate on this item by providing additional information not in the ms, or briefly explain why the item is not applicable/relevant for your study

Data Collection
Two NPs who were not directly involved in MDRs and patient care were trained to audit the processes. The two NPs have 8 and 6 years of ICU experience, respectively. The two observers were temporarily exempted from clinical work during the study period. To ensure adequate training in the study methodology, personnel piloted data collection and evaluation of communication accuracy were performed by the two observers and supervised by the senior investigator (C.H.L.) during a 4-week run-in period. The two observers performed in-field observation and audio recordings and audited the process together using a standardized form (Supplementary Table S2 in Multimedia Appendix 3) to reduce the possibility of losing any useful information and ensure the correct assessment. Before MDRs, observers measured the amount of time for pre-rounding data gathering by the NP using the built-in stopwatch app on mobile phones. Subsequently, observers arrived before the start of MDRs and refrained from participating in the discussion during MDRs. Baseline data of patients, information on patient disease severity and therapeutic interventions during MDRs and established care

4a-iii) Information giving during recruitment

Information given during recruitment. Specify how participants were briefed for recruitment and in the informed consent procedures (e.g., publish the informed consent documentation as appendix, see also item X26), as this information may have an effect on user self-selection, user expectation and may also bias results.

	1	2	3	4	5	
subitem not at all important	○	○	◉	○	○	essential

清除選取的項目

Does your paper address subitem 4a-iii?

Copy and paste relevant sections from the manuscript (include quotes in quotation marks "like this" to indicate direct quotes from your manuscript), or elaborate on this item by providing additional information not in the ms, or briefly explain why the item is not applicable/relevant for your study

To ensure effective implementation of i-Dashboard, we prepared education materials (Multimedia Appendix 2) in the form of brief presentations for all the healthcare providers working in study units.

4b) Settings and locations where the data were collected

Does your paper address CONSORT subitem 4b? *

Copy and paste relevant sections from the manuscript (include quotes in quotation marks "like this" to indicate direct quotes from your manuscript), or elaborate on this item by providing additional information not in the ms, or briefly explain why the item is not applicable/relevant for your study

The study was conducted in a 1300-bed university hospital that offers first-line and tertiary referral services for a population of approximately 1.8 million in southern Taiwan. A cluster randomized trial was conducted in two of the four surgical ICUs with 10 and 8 beds.

4b-i) Report if outcomes were (self-)assessed through online questionnaires

Clearly report if outcomes were (self-)assessed through online questionnaires (as common in web-based trials) or otherwise.

	1	2	3	4	5	
subitem not at all important	◉	○	○	○	○	essential

清除選取的項目

Does your paper address subitem 4b-i? *

Copy and paste relevant sections from the manuscript (include quotes in quotation marks "like this" to indicate direct quotes from your manuscript), or elaborate on this item by providing additional information not in the ms, or briefly explain why the item is not applicable/relevant for your study

Not applicable in the present study

4b-ii) Report how institutional affiliations are displayed

Report how institutional affiliations are displayed to potential participants [on ehealth media], as affiliations with prestigious hospitals or universities may affect volunteer rates, use, and reactions with regards to an intervention.(Not a required item – describe only if this may bias results)

	1	2	3	4	5	
subitem not at all important	○	○	○	○	◉	essential

清除選取的項目

Does your paper address subitem 4b-ii?

Copy and paste relevant sections from the manuscript (include quotes in quotation marks "like this" to indicate direct quotes from your manuscript), or elaborate on this item by providing additional information not in the ms, or briefly explain why the item is not applicable/relevant for your study

The study was conducted in a 1300-bed university hospital that offers first-line and tertiary referral services for a population of approximately 1.8 million in southern Taiwan.

5) The interventions for each group with sufficient details to allow replication, including how and when they were actually administered

5-i) Mention names, credential, affiliations of the developers, sponsors, and owners

Mention names, credential, affiliations of the developers, sponsors, and owners [6] (if authors/evaluators are owners or developer of the software, this needs to be declared in a “Conflict of interest” section or mentioned elsewhere in the manuscript).

	1	2	3	4	5	
subitem not at all important	○	○	○	○	◉	essential

清除選取的項目

Does your paper address subitem 5-i?

Copy and paste relevant sections from the manuscript (include quotes in quotation marks "like this" to indicate direct quotes from your manuscript), or elaborate on this item by providing additional information not in the ms, or briefly explain why the item is not applicable/relevant for your study

Established EMR
The established EMR environment applied in the ICU includes the Philips IntelliSpace Critical Care & Anesthesia information system (ICCA; Philips, Eindhoven, Netherlands) and Hospital Information System (HIS) developed by the institutional Department of Information Technology and its sub-systems, including Laboratory Information System (LIS) and Picture Archiving and Communication System (PACS). Philips ICCA is an ICU-specific EMR system that provides information essential for critical care [17]. Patient data are organized by data category (demographics, vital signs, laboratory data, etc.) in a series of tabs or window panels. HIS supports text data key-in and patient order entry.
Development and Architecture of i-Dashboard
This study evaluated the performance of the first version of i-Dashboard. The i-Dashboard (Advantech, Taipei, Taiwan) was custom-developed under the guidance of multidisciplinary professionals, including physicians, registered nurses, NPs, RTs, pharmacists and dietitians, rather than physicians only because information for MDRs needs may vary based on the clinical role. Every healthcare provider working in the surgical ICU participated in developing i-Dashboard. Different professionals held preparatory meetings of their own. Before i-Dashboard was formally implemented for clinical use, the structure and layout were repeatedly revised and tailored to achieve broad acceptance among directors of multidisciplinary providers in the ICU.
The architecture of i-Dashboard is summarized in Multimedia Appendix 2. In the static mode, the i-Dashboard was designed to substitute as a station whiteboard with lists of patients, information to aid emergency evacuation and on-duty healthcare physicians, nurses and NPs. Patient-level data in i-Dashboard were modified from the MDR checklist and digitally transformed. Therefore, data were pre-identified and retrieved from different origins in the established EMR environment, especially ICCA. Instead of database-centered displays, these data were reorganized based on the requirements of different professionals/disciplines to form dashboard pages (i.e., an overview page and a RT-pharmacist-dietitian page) and element blocks. In addition to colored signaling for values outside the reference ranges, i-Dashboard was designed to support built-in automated calculation of severity scores (e.g., Simplified Acute Physiology Score II [SAPS II] and Sequential Organ Failure Assessment [SOFA]) and visualization of time-series data to expedite navigation of patient condition. Time-series data (e.g., vital signs, laboratory data or severity scores) that were transformed into line charts as needed can be accessed through the hyperlinks located at left upper corner of element blocks on the overview page. The page of time-series data was designed to support both fixed (e.g., last 24 hours or last 3 days) and relative custom time frames are available.
The technical details behind i-Dashboard are summarized in Figure 1. We used a Windows 10 desktop as the visualization platform to support i-dashboard. A K8s-based WISE-PaaS 4.0 platform (Advantech) facilitates the integration of diverse devices and communication protocols, making data exchange and system development agile. The entire platform was developed and deployed on 6 VMware servers, each with a 256-GB hard drive and 32-GB memory, and a 24-core Intel Xeon Gold 6248R 3.00 GHz processor. The servers received the data from the Philips ICCA, HIS, LIS database servers.
In the study units, both the established EMR and i-Dashboard can be accessed through desktop computers with 17- or 22-inch monitors or mobile platforms.

5-ii) Describe the history/development process

Describe the history/development process of the application and previous formative evaluations (e.g., focus groups, usability testing), as these will have an impact on adoption/use rates and help with interpreting results.

	1	2	3	4	5	
subitem not at all important	○	○	○	○	◉	essential

清除選取的項目

Does your paper address subitem 5-ii?

Copy and paste relevant sections from the manuscript (include quotes in quotation marks "like this" to indicate direct quotes from your manuscript), or elaborate on this item by providing additional information not in the ms, or briefly explain why the item is not applicable/relevant for your study

Development and Architecture of i-Dashboard
This study evaluated the performance of the first version of i-Dashboard. The i-Dashboard (Advantech, Taipei, Taiwan) was custom-developed under the guidance of multidisciplinary professionals, including physicians, registered nurses, NPs, RTs, pharmacists and dietitians, rather than physicians only because information for MDRs needs may vary based on the clinical role. Every healthcare provider working in the surgical ICU participated in developing i-Dashboard. Different professionals held preparatory meetings of their own. Before i-Dashboard was formally implemented for clinical use, the structure and layout were repeatedly revised and tailored to achieve broad acceptance among directors of multidisciplinary providers in the ICU.
The architecture of i-Dashboard is summarized in Multimedia Appendix 2. In the static mode, the i-Dashboard was designed to substitute as a station whiteboard with lists of patients, information to aid emergency evacuation and on-duty healthcare physicians, nurses and NPs. Patient-level data in i-Dashboard were modified from the MDR checklist and digitally transformed. Therefore, data were pre-identified and retrieved from different origins in the established EMR environment, especially ICCA. Instead of database-centered displays, these data were reorganized based on the requirements of different professionals/disciplines to form dashboard pages (i.e., an overview page and a RT-pharmacist-dietitian page) and element blocks. In addition to colored signaling for values outside the reference ranges, i-Dashboard was designed to support built-in automated calculation of severity scores (e.g., Simplified Acute Physiology Score II [SAPS II] and Sequential Organ Failure Assessment [SOFA]) and visualization of time-series data to expedite navigation of patient condition. Time-series data (e.g., vital signs, laboratory data or severity scores) that were transformed into line charts as needed can be accessed through the hyperlinks located at left upper corner of element blocks on the overview page. The page of time-series data was designed to support both fixed (e.g., last 24 hours or last 3 days) and relative custom time frames are available.
The technical details behind i-Dashboard are summarized in Figure 1. We used a Windows 10 desktop as the visualization platform to support i-dashboard. A K8s-based WISE-PaaS 4.0 platform (Advantech) facilitates the integration of diverse devices and communication protocols, making data exchange and system development agile. The entire platform was developed and deployed on 6 VMware servers, each with a 256-GB hard drive and 32-GB memory, and a 24-core Intel Xeon Gold 6248R 3.00 GHz processor. The servers received the data from the Philips ICCA, HIS, LIS database servers.
In the study units, both the established EMR and i-Dashboard can be accessed through desktop computers with 17- or 22-inch monitors or mobile platforms.

5-iii) Revisions and updating

Revisions and updating. Clearly mention the date and/or version number of the application/intervention (and comparator, if applicable) evaluated, or describe whether the intervention underwent major changes during the evaluation process, or whether the development and/or content was “frozen” during the trial. Describe dynamic components such as news feeds or changing content which may have an impact on the replicability of the intervention (for unexpected events see item 3b).

	1	2	3	4	5	
subitem not at all important	◉	○	○	○	○	essential

清除選取的項目

Does your paper address subitem 5-iii?

Copy and paste relevant sections from the manuscript (include quotes in quotation marks "like this" to indicate direct quotes from your manuscript), or elaborate on this item by providing additional information not in the ms, or briefly explain why the item is not applicable/relevant for your study

Not applicable in the present study (the development was frozen during the trial but would be started after the trial)

5-iv) Quality assurance methods

Provide information on quality assurance methods to ensure accuracy and quality of information provided [1], if applicable.

	1	2	3	4	5	
subitem not at all important	◉	○	○	○	○	essential

清除選取的項目

Does your paper address subitem 5-iv?

Copy and paste relevant sections from the manuscript (include quotes in quotation marks "like this" to indicate direct quotes from your manuscript), or elaborate on this item by providing additional information not in the ms, or briefly explain why the item is not applicable/relevant for your study

Not applicable in the present study

5-v) Ensure replicability by publishing the source code, and/or providing screenshots/screen-capture video, and/or providing flowcharts of the algorithms used

Ensure replicability by publishing the source code, and/or providing screenshots/screen-capture video, and/or providing flowcharts of the algorithms used. Replicability (i.e., other researchers should in principle be able to replicate the study) is a hallmark of scientific reporting.

	1	2	3	4	5	
subitem not at all important	○	○	○	○	◉	essential

清除選取的項目

Does your paper address subitem 5-v?

Copy and paste relevant sections from the manuscript (include quotes in quotation marks "like this" to indicate direct quotes from your manuscript), or elaborate on this item by providing additional information not in the ms, or briefly explain why the item is not applicable/relevant for your study

The architecture of i-Dashboard is summarized in Multimedia Appendix 2. Screenshots of i-Dashboard were provided.

5-vi) Digital preservation

Digital preservation: Provide the URL of the application, but as the intervention is likely to change or disappear over the course of the years; also make sure the intervention is archived (Internet Archive, webcitation.org, and/or publishing the source code or screenshots/videos alongside the article). As pages behind login screens cannot be archived, consider creating demo pages which are accessible without login.

	1	2	3	4	5	
subitem not at all important	○	○	○	○	◉	essential

清除選取的項目

Does your paper address subitem 5-vi?

Copy and paste relevant sections from the manuscript (include quotes in quotation marks "like this" to indicate direct quotes from your manuscript), or elaborate on this item by providing additional information not in the ms, or briefly explain why the item is not applicable/relevant for your study

The architecture of i-Dashboard is summarized in Multimedia Appendix 2. Screenshots of i-Dashboard were provided.

5-vii) Access

Access: Describe how participants accessed the application, in what setting/context, if they had to pay (or were paid) or not, whether they had to be a member of specific group. If known, describe how participants obtained “access to the platform and Internet” [1]. To ensure access for editors/reviewers/readers, consider to provide a “backdoor” login account or demo mode for reviewers/readers to explore the application (also important for archiving purposes, see vi).

	1	2	3	4	5	
subitem not at all important	○	◉	○	○	○	essential

清除選取的項目

Does your paper address subitem 5-vii? *

Copy and paste relevant sections from the manuscript (include quotes in quotation marks "like this" to indicate direct quotes from your manuscript), or elaborate on this item by providing additional information not in the ms, or briefly explain why the item is not applicable/relevant for your study

Not applicable in the present study

5-viii) Mode of delivery, features/functionalities/components of the intervention and comparator, and the theoretical framework

Describe mode of delivery, features/functionalities/components of the intervention and comparator, and the theoretical framework [6] used to design them (instructional strategy [1], behaviour change techniques, persuasive features, etc., see e.g., [7, 8] for terminology). This includes an in-depth description of the content (including where it is coming from and who developed it) [1]," whether [and how] it is tailored to individual circumstances and allows users to track their progress and receive feedback" [6]. This also includes a description of communication delivery channels and – if computer-mediated communication is a component – whether communication was synchronous or asynchronous [6]. It also includes information on presentation strategies [1], including page design principles, average amount of text on pages, presence of hyperlinks to other resources, etc. [1].

	1	2	3	4	5	
subitem not at all important	○	○	○	○	◉	essential

清除選取的項目

Does your paper address subitem 5-viii? *

Copy and paste relevant sections from the manuscript (include quotes in quotation marks "like this" to indicate direct quotes from your manuscript), or elaborate on this item by providing additional information not in the ms, or briefly explain why the item is not applicable/relevant for your study

In the study hospital, both the established EMR and i-Dashboard can be accessed through desktop computers with 17- or 22-inch monitors or mobile platforms.

5-ix) Describe use parameters

Describe use parameters (e.g., intended "doses" and optimal timing for use). Clarify what instructions or recommendations were given to the user, e.g., regarding timing, frequency, heaviness of use, if any, or was the intervention used ad libitum.

	1	2	3	4	5	
subitem not at all important	○	○	○	○	◉	essential

清除選取的項目

Does your paper address subitem 5-ix?

Copy and paste relevant sections from the manuscript (include quotes in quotation marks "like this" to indicate direct quotes from your manuscript), or elaborate on this item by providing additional information not in the ms, or briefly explain why the item is not applicable/relevant for your study

The established EMR (control) and i-Dashboard (intervention) were randomly assigned as tools to facilitate pre-rounding information collection and MDRs in the two units and exchanged at two-week intervals.
Before this trial, MDRs have been carried out in the study units for ~5 years. An integrated multidisciplinary care team is comprised of at least an intensivist, a registered nurse, a NP, a RT, a pharmacist and a dietitian. All these providers attend MDRs held on a regular schedule three times a week. MDRs are conducted only for patients who stay for more than 7 days because patients receiving surgical ICU care for more than 7 days have a high rate of in-hospital mortality [15], and ~75% of patients have a length of stay for 7 days or less in study units.

5-x) Clarify the level of human involvement

Clarify the level of human involvement (care providers or health professionals, also technical assistance) in the e-intervention or as co-intervention (detail number and expertise of professionals involved, if any, as well as "type of assistance offered, the timing and frequency of the support, how it is initiated, and the medium by which the assistance is delivered". It may be necessary to distinguish between the level of human involvement required for the trial, and the level of human involvement required for a routine application outside of a RCT setting (discuss under item 21 – generalizability).

	1	2	3	4	5	
subitem not at all important	○	○	○	○	◉	essential

清除選取的項目

Does your paper address subitem 5-x?

Copy and paste relevant sections from the manuscript (include quotes in quotation marks "like this" to indicate direct quotes from your manuscript), or elaborate on this item by providing additional information not in the ms, or briefly explain why the item is not applicable/relevant for your study

Before this trial, MDRs have been carried out in the study units for ~5 years. An integrated multidisciplinary care team is comprised of at least an intensivist, a registered nurse, a NP, a RT, a pharmacist and a dietitian. All these providers attend MDRs held on a regular schedule three times a week. MDRs are conducted only for patients who stay for more than 7 days because patients receiving surgical ICU care for more than 7 days have a high rate of in-hospital mortality [15], and ~75% of patients have a length of stay for 7 days or less in study units.

5-xi) Report any prompts/reminders used

Report any prompts/reminders used: Clarify if there were prompts (letters, emails, phone calls, SMS) to use the application, what triggered them, frequency etc. It may be necessary to distinguish between the level of prompts/reminders required for the trial, and the level of prompts/reminders for a routine application outside of a RCT setting (discuss under item 21 – generalizability).

	1	2	3	4	5	
subitem not at all important	◉	○	○	○	○	essential

清除選取的項目

Does your paper address subitem 5-xi? *

Copy and paste relevant sections from the manuscript (include quotes in quotation marks "like this" to indicate direct quotes from your manuscript), or elaborate on this item by providing additional information not in the ms, or briefly explain why the item is not applicable/relevant for your study

Not applicable in the present study

5-xii) Describe any co-interventions (incl. training/support)

Describe any co-interventions (incl. training/support): Clearly state any interventions that are provided in addition to the targeted eHealth intervention, as ehealth intervention may not be designed as stand-alone intervention. This includes training sessions and support [1]. It may be necessary to distinguish between the level of training required for the trial, and the level of training for a routine application outside of a RCT setting (discuss under item 21 – generalizability.

	1	2	3	4	5	
subitem not at all important	◉	○	○	○	○	essential

清除選取的項目

Does your paper address subitem 5-xii? *

Copy and paste relevant sections from the manuscript (include quotes in quotation marks "like this" to indicate direct quotes from your manuscript), or elaborate on this item by providing additional information not in the ms, or briefly explain why the item is not applicable/relevant for your study

To ensure effective implementation of i-Dashboard, we prepared education materials (Multimedia Appendix 2) in the form of brief presentations for all the healthcare providers working in study units. Subsequently, the use of i-Dashboard was tested consistently for 4 weeks.

6a) Completely defined pre-specified primary and secondary outcome measures, including how and when they were assessed

Does your paper address CONSORT subitem 6a? *

Copy and paste relevant sections from the manuscript (include quotes in quotation marks "like this" to indicate direct quotes from your manuscript), or elaborate on this item by providing additional information not in the ms, or briefly explain why the item is not applicable/relevant for your study

Primary outcomes of interest included time of data gathering before MDRs and communication accuracy during MDRs. The amount of time that NPs spent gathering clinical data before rounding was recorded. Communication accuracy was evaluated based on the items listed on the standardized form (Supplementary Table S2 in Multimedia Appendix 3) through direct field observation and audio recordings. Spoken information was compared with EMR data captured by screenshots taken prior to patient presentations. Data communication, including laboratory and non-laboratory data, was considered inaccurate (i.e., data misrepresentation) when the values or data were not correctly reported. For laboratory data communication, only abnormal laboratory data points (outside the reference ranges) were assessed. Laboratory misrepresentations were further classified into several categories as previously defined [8], including omission, old data, pending results, misinterpretation and erroneous values. Secondary outcomes included information exchange, using effective recommendations initiated by RTs, pharmacists and dietitians as an index, and clinical satisfaction on i-Dashboard. The recommendations initiated by RTs, pharmacists and dietitians, as exemplified in Supplementary Table S3 in Multimedia Appendix 3, were considered effective on the condition that they were successfully adopted into the care plan documented on EMR. Clinical satisfaction with i-Dashboard as an information management tool was investigated using Likert-scale questionnaires (Multimedia Appendix 4) from previously validated survey instruments with minor modifications [12]. Questionnaire 1 was designed to capture the perceived efficiency, accuracy, and safety of the established EMR and implemented i-Dashboard when used to prepare for data gathering and assist MDRs. Questionnaire 2 was designed to identify intention to use and personal impact of i-Dashboard as a result of i-Dashboard implementation. At the immediate end of the study, the two surveys were administered in hard copy form to study participants. Each participant responded only once.

6a-i) Online questionnaires: describe if they were validated for online use and apply CHERRIES items to describe how the questionnaires were designed/deployed

If outcomes were obtained through online questionnaires, describe if they were validated for online use and apply CHERRIES items to describe how the questionnaires were designed/deployed [9].

	1	2	3	4	5	
subitem not at all important	◉	○	○	○	○	essential

清除選取的項目

Does your paper address subitem 6a-i?

Copy and paste relevant sections from manuscript text

Not applicable in the present study

6a-ii) Describe whether and how "use" (including intensity of use/dosage) was defined/measured/monitored

Describe whether and how "use" (including intensity of use/dosage) was defined/measured/monitored (logins, logfile analysis, etc.). Use/adoption metrics are important process outcomes that should be reported in any ehealth trial.

	1	2	3	4	5	
subitem not at all important	◉	○	○	○	○	essential

清除選取的項目

Does your paper address subitem 6a-ii?

Copy and paste relevant sections from manuscript text

Not applicable in the present study

6a-iii) Describe whether, how, and when qualitative feedback from participants was obtained

Describe whether, how, and when qualitative feedback from participants was obtained (e.g., through emails, feedback forms, interviews, focus groups).

	1	2	3	4	5	
subitem not at all important	○	○	○	○	◉	essential

清除選取的項目

Does your paper address subitem 6a-iii?

Copy and paste relevant sections from manuscript text

Clinical satisfaction with i-Dashboard as an information management tool was investigated using Likert-scale questionnaires (Multimedia Appendix 4) from previously validated survey instruments with minor modifications [12]. Questionnaire 1 was designed to capture the perceived efficiency, accuracy, and safety of the established EMR and implemented i-Dashboard when used to prepare for data gathering and assist MDRs. Questionnaire 2 was designed to identify intention to use and personal impact of i-Dashboard as a result of i-Dashboard implementation. At the immediate end of the study, the two surveys were administered in hard copy form to study participants. Each participant responded only once.

6b) Any changes to trial outcomes after the trial commenced, with reasons

Does your paper address CONSORT subitem 6b? *

Copy and paste relevant sections from the manuscript (include quotes in quotation marks "like this" to indicate direct quotes from your manuscript), or elaborate on this item by providing additional information not in the ms, or briefly explain why the item is not applicable/relevant for your study

Not applicable in the present study

7a) How sample size was determined

NPT: When applicable, details of whether and how the clustering by care provides or centers was addressed

7a-i) Describe whether and how expected attrition was taken into account when calculating the sample size

Describe whether and how expected attrition was taken into account when calculating the sample size.

	1	2	3	4	5	
subitem not at all important	○	○	○	○	◉	essential

清除選取的項目

Does your paper address subitem 7a-i?

Copy and paste relevant sections from manuscript title (include quotes in quotation marks "like this" to indicate direct quotes from your manuscript), or elaborate on this item by providing additional information not in the ms, or briefly explain why the item is not applicable/relevant for your study

Before the trial began, it was estimated that 12 MDRs would be observed per unit in a 2-week period. To estimate the required sample size, we used a cluster randomized trial design to account for the positive intraclass correlation expected among members of the same group or cluster. Observational pilot data for pre-rounding data gathering were collected. The results showed that the mean time difference between the use of the established EMR and i-Dashboard was 3 minutes with a variance of 4. The intraclass correlation coefficient of this pilot study was 0.47. The corresponding estimations were treated as true parameters. Thus, enrolling approximately 144 patients during 24 unit-weeks would provide a power of 90% at a type I error rate of 0.05 to detect an intervention effect of 3 minutes between groups.

7b) When applicable, explanation of any interim analyses and stopping guidelines

Does your paper address CONSORT subitem 7b? *

Copy and paste relevant sections from the manuscript (include quotes in quotation marks "like this" to indicate direct quotes from your manuscript), or elaborate on this item by providing additional information not in the ms, or briefly explain why the item is not applicable/relevant for your study

Not applicable in the present study

8a) Method used to generate the random allocation sequence

NPT: When applicable, how care providers were allocated to each trial group

Does your paper address CONSORT subitem 8a? *

Copy and paste relevant sections from the manuscript (include quotes in quotation marks "like this" to indicate direct quotes from your manuscript), or elaborate on this item by providing additional information not in the ms, or briefly explain why the item is not applicable/relevant for your study

Not applicable in the present study

8b) Type of randomisation; details of any restriction (such as blocking and block size)

Does your paper address CONSORT subitem 8b? *

Copy and paste relevant sections from the manuscript (include quotes in quotation marks "like this" to indicate direct quotes from your manuscript), or elaborate on this item by providing additional information not in the ms, or briefly explain why the item is not applicable/relevant for your study

A cluster randomized trial was conducted in two of the four surgical ICUs with 10 and 8 beds. The established EMR (control) and i-Dashboard (intervention) were randomly assigned as tools to facilitate pre-rounding information collection and MDRs in the two units and exchanged at two-week intervals.

9) Mechanism used to implement the random allocation sequence (such as sequentially numbered containers), describing any steps taken to conceal the sequence until interventions were assigned

Does your paper address CONSORT subitem 9? *

Copy and paste relevant sections from the manuscript (include quotes in quotation marks "like this" to indicate direct quotes from your manuscript), or elaborate on this item by providing additional information not in the ms, or briefly explain why the item is not applicable/relevant for your study

Not applicable in the present study

10) Who generated the random allocation sequence, who enrolled participants, and who assigned participants to interventions

Does your paper address CONSORT subitem 10? *

Copy and paste relevant sections from the manuscript (include quotes in quotation marks "like this" to indicate direct quotes from your manuscript), or elaborate on this item by providing additional information not in the ms, or briefly explain why the item is not applicable/relevant for your study

The co-auther and statistician, Pei-Fang Su, generated the random allocation sequence.

11a) If done, who was blinded after assignment to interventions (for example, participants, care providers, those assessing outcomes) and how

NPT: Whether or not administering co-interventions were blinded to group assignment

11a-i) Specify who was blinded, and who wasn't

Specify who was blinded, and who wasn't. Usually, in web-based trials it is not possible to blind the participants [1, 3] (this should be clearly acknowledged), but it may be possible to blind outcome assessors, those doing data analysis or those administering co-interventions (if any).

	1	2	3	4	5	
subitem not at all important	◉	○	○	○	○	essential

清除選取的項目

Does your paper address subitem 11a-i? *

Copy and paste relevant sections from the manuscript (include quotes in quotation marks "like this" to indicate direct quotes from your manuscript), or elaborate on this item by providing additional information not in the ms, or briefly explain why the item is not applicable/relevant for your study

Not applicable in the present study

11a-ii) Discuss e.g., whether participants knew which intervention was the "intervention of interest" and which one was the "comparator"

Informed consent procedures (4a-ii) can create biases and certain expectations - discuss e.g., whether participants knew which intervention was the "intervention of interest" and which one was the "comparator".

	1	2	3	4	5	
subitem not at all important	◉	○	○	○	○	essential

清除選取的項目

Does your paper address subitem 11a-ii?

Copy and paste relevant sections from the manuscript (include quotes in quotation marks "like this" to indicate direct quotes from your manuscript), or elaborate on this item by providing additional information not in the ms, or briefly explain why the item is not applicable/relevant for your study

Not applicable in the present study

11b) If relevant, description of the similarity of interventions

(this item is usually not relevant for ehealth trials as it refers to similarity of a placebo or sham intervention to a active medication/intervention)

Does your paper address CONSORT subitem 11b? *

Copy and paste relevant sections from the manuscript (include quotes in quotation marks "like this" to indicate direct quotes from your manuscript), or elaborate on this item by providing additional information not in the ms, or briefly explain why the item is not applicable/relevant for your study

Not applicable in the present study

12a) Statistical methods used to compare groups for primary and secondary outcomes

NPT: When applicable, details of whether and how the clustering by care providers or centers was addressed

Does your paper address CONSORT subitem 12a? *

Copy and paste relevant sections from the manuscript (include quotes in quotation marks "like this" to indicate direct quotes from your manuscript), or elaborate on this item by providing additional information not in the ms, or briefly explain why the item is not applicable/relevant for your study

Categorical variables were analyzed using the $\chi 2$ test or Fisher's exact test as needed. Continuous variables and Likert scale were analyzed using the Mann-Whitney U test. Statistical analyses were performed using R software (Version 3.4.3; Foundation for Statistical Computing, Vienna, Austria). A two-tailed P<.05 was considered statistically significant.

12a-i) Imputation techniques to deal with attrition / missing values

Imputation techniques to deal with attrition / missing values: Not all participants will use the intervention/comparator as intended and attrition is typically high in ehealth trials. Specify how participants who did not use the application or dropped out from the trial were treated in the statistical analysis (a complete case analysis is strongly discouraged, and simple imputation techniques such as LOCF may also be problematic [4]).

	1	2	3	4	5	
subitem not at all important	◉	○	○	○	○	essential

清除選取的項目

Does your paper address subitem 12a-i? *

Copy and paste relevant sections from the manuscript (include quotes in quotation marks "like this" to indicate direct quotes from your manuscript), or elaborate on this item by providing additional information not in the ms, or briefly explain why the item is not applicable/relevant for your study

Not applicable in the present study

12b) Methods for additional analyses, such as subgroup analyses and adjusted analyses

Does your paper address CONSORT subitem 12b? *

Copy and paste relevant sections from the manuscript (include quotes in quotation marks "like this" to indicate direct quotes from your manuscript), or elaborate on this item by providing additional information not in the ms, or briefly explain why the item is not applicable/relevant for your study

Not applicable in the present study

X26) REB/IRB Approval and Ethical Considerations [recommended as subheading under "Methods"] (not a CONSORT item)

X26-i) Comment on ethics committee approval

	1	2	3	4	5	
subitem not at all important	○	○	○	○	◉	essential

清除選取的項目

Does your paper address subitem X26-i?

Copy and paste relevant sections from the manuscript (include quotes in quotation marks "like this" to indicate direct quotes from your manuscript), or elaborate on this item by providing additional information not in the ms, or briefly explain why the item is not applicable/relevant for your study

The study protocol was approved by the Institutional Review Board of National Cheng Kung University Hospital (B-ER-110-040).

x26-ii) Outline informed consent procedures

Outline informed consent procedures e.g., if consent was obtained offline or online (how? Checkbox, etc.?), and what information was provided (see 4a-ii). See [6] for some items to be included in informed consent documents.

	1	2	3	4	5	
subitem not at all important	○	○	○	○	◉	essential

清除選取的項目

Does your paper address subitem X26-ii?

Copy and paste relevant sections from the manuscript (include quotes in quotation marks "like this" to indicate direct quotes from your manuscript), or elaborate on this item by providing additional information not in the ms, or briefly explain why the item is not applicable/relevant for your study

Informed consent was obtained from all participants in the study.

X26-iii) Safety and security procedures

Safety and security procedures, incl. privacy considerations, and any steps taken to reduce the likelihood or detection of harm (e.g., education and training, availability of a hotline)

	1	2	3	4	5	
subitem not at all important	◉	○	○	○	○	essential

清除選取的項目

Does your paper address subitem X26-iii?

Copy and paste relevant sections from the manuscript (include quotes in quotation marks "like this" to indicate direct quotes from your manuscript), or elaborate on this item by providing additional information not in the ms, or briefly explain why the item is not applicable/relevant for your study

Not applicable in the present study

RESULTS

13a) For each group, the numbers of participants who were randomly assigned, received intended treatment, and were analysed for the primary outcome

NPT: The number of care providers or centers performing the intervention in each group and the number of patients treated by each care provider in each center

Does your paper address CONSORT subitem 13a? *

Copy and paste relevant sections from the manuscript (include quotes in quotation marks "like this" to indicate direct quotes from your manuscript), or elaborate on this item by providing additional information not in the ms, or briefly explain why the item is not applicable/relevant for your study

Between April 26, 2021, and July 18, 2021, there were 173 admissions to the two study units. A total of 90 multidisciplinary providers (Table 1) participated in MDRs for the 25 individual patients (Table 2); 78 MDRs were performed with the established EMR environment, whereas 91 MDRs were performed with i-Dashboard (Figure 3).

13b) For each group, losses and exclusions after randomisation, together with reasons

Does your paper address CONSORT subitem 13b? (NOTE: Preferably, this is shown in a CONSORT flow diagram) *

Copy and paste relevant sections from the manuscript (include quotes in quotation marks "like this" to indicate direct quotes from your manuscript), or elaborate on this item by providing additional information not in the ms, or briefly explain why the item is not applicable/relevant for your study

Not applicable in the present study

13b-i) Attrition diagram

Strongly recommended: An attrition diagram (e.g., proportion of participants still logging in or using the intervention/comparator in each group plotted over time, similar to a survival curve) or other figures or tables demonstrating usage/dose/engagement.

	1	2	3	4	5	
subitem not at all important	(●)	○	○	○	○	essential

清除選取的項目

Does your paper address subitem 13b-i?

Copy and paste relevant sections from the manuscript or cite the figure number if applicable (include quotes in quotation marks "like this" to indicate direct quotes from your manuscript), or elaborate on this item by providing additional information not in the ms, or briefly explain why the item is not applicable/relevant for your study

Not applicable in the present study because this study was performed in ICUs

14a) Dates defining the periods of recruitment and follow-up

Does your paper address CONSORT subitem 14a? *

Copy and paste relevant sections from the manuscript (include quotes in quotation marks "like this" to indicate direct quotes from your manuscript), or elaborate on this item by providing additional information not in the ms, or briefly explain why the item is not applicable/relevant for your study

Not applicable in the present study

14a-i) Indicate if critical “secular events” fell into the study period

Indicate if critical “secular events” fell into the study period, e.g., significant changes in Internet resources available or “changes in computer hardware or Internet delivery resources”

	1	2	3	4	5	
subitem not at all important	(●)	( )	( )	( )	( )	essential

清除選取的項目

Does your paper address subitem 14a-i?

Copy and paste relevant sections from the manuscript (include quotes in quotation marks "like this" to indicate direct quotes from your manuscript), or elaborate on this item by providing additional information not in the ms, or briefly explain why the item is not applicable/relevant for your study

Not applicable in the present study

14b) Why the trial ended or was stopped (early)

Does your paper address CONSORT subitem 14b? *

Copy and paste relevant sections from the manuscript (include quotes in quotation marks "like this" to indicate direct quotes from your manuscript), or elaborate on this item by providing additional information not in the ms, or briefly explain why the item is not applicable/relevant for your study

Not applicable in the present study

15) A table showing baseline demographic and clinical characteristics for each group

NPT: When applicable, a description of care providers (case volume, qualification, expertise, etc.) and centers (volume) in each group

Does your paper address CONSORT subitem 15? *

Copy and paste relevant sections from the manuscript (include quotes in quotation marks "like this" to indicate direct quotes from your manuscript), or elaborate on this item by providing additional information not in the ms, or briefly explain why the item is not applicable/relevant for your study

Table 3 shows baseline demographic and clinical characteristics for each group.

15-i) Report demographics associated with digital divide issues

In ehealth trials it is particularly important to report demographics associated with digital divide issues, such as age, education, gender, social-economic status, computer/Internet/ehealth literacy of the participants, if known.

	1	2	3	4	5	
subitem not at all important	○	○	○	○	◉	essential

清除選取的項目

Does your paper address subitem 15-i? *

Copy and paste relevant sections from the manuscript (include quotes in quotation marks "like this" to indicate direct quotes from your manuscript), or elaborate on this item by providing additional information not in the ms, or briefly explain why the item is not applicable/relevant for your study

Table 1 reports demographics of participants, including age, gender and experiences.

16) For each group, number of participants (denominator) included in each analysis and whether the analysis was by original assigned groups

16-i) Report multiple “denominators” and provide definitions

Report multiple “denominators” and provide definitions: Report N’s (and effect sizes) “across a range of study participation [and use] thresholds” [1], e.g., N exposed, N consented, N used more than x times, N used more than y weeks, N participants “used” the intervention/comparator at specific pre-defined time points of interest (in absolute and relative numbers per group). Always clearly define “use” of the intervention.

	1	2	3	4	5	
subitem not at all important	○	○	○	○	◉	essential

清除選取的項目

Does your paper address subitem 16-i? *

Copy and paste relevant sections from the manuscript (include quotes in quotation marks "like this" to indicate direct quotes from your manuscript), or elaborate on this item by providing additional information not in the ms, or briefly explain why the item is not applicable/relevant for your study

N is clearly described in the paper and Tables.

16-ii) Primary analysis should be intent-to-treat

Primary analysis should be intent-to-treat, secondary analyses could include comparing only “users”, with the appropriate caveats that this is no longer a randomized sample (see 18-i).

	1	2	3	4	5	
subitem not at all important	○	○	○	○	◉	essential

清除選取的項目

Does your paper address subitem 16-ii?

Copy and paste relevant sections from the manuscript (include quotes in quotation marks "like this" to indicate direct quotes from your manuscript), or elaborate on this item by providing additional information not in the ms, or briefly explain why the item is not applicable/relevant for your study

Primary analysis was intent-to-treat.

17a) For each primary and secondary outcome, results for each group, and the estimated effect size and its precision (such as 95% confidence interval)

Does your paper address CONSORT subitem 17a? *

Copy and paste relevant sections from the manuscript (include quotes in quotation marks "like this" to indicate direct quotes from your manuscript), or elaborate on this item by providing additional information not in the ms, or briefly explain why the item is not applicable/relevant for your study

The median (interquartile range [IQR]) time for pre-rounding data gathering was 10.4 (9.1-11.8) and 4.6 (3.5-5.8) minutes per patient using the established EMR and i-Dashboard (P<.001; Table 3), respectively , indicating a reduction of 5.8 (95% confidence interval 5.2-6.4) minutes when using i-Dashboard.

17a-i) Presentation of process outcomes such as metrics of use and intensity of use

In addition to primary/secondary (clinical) outcomes, the presentation of process outcomes such as metrics of use and intensity of use (dose, exposure) and their operational definitions is critical. This does not only refer to metrics of attrition (13-b) (often a binary variable), but also to more continuous exposure metrics such as “average session length”. These must be accompanied by a technical description how a metric like a “session” is defined (e.g., timeout after idle time) [1] (report under item 6a).

	1	2	3	4	5	
subitem not at all important	◉	○	○	○	○	essential

清除選取的項目

Does your paper address subitem 17a-i?

Copy and paste relevant sections from the manuscript (include quotes in quotation marks "like this" to indicate direct quotes from your manuscript), or elaborate on this item by providing additional information not in the ms, or briefly explain why the item is not applicable/relevant for your study

Not applicable in the present study

17b) For binary outcomes, presentation of both absolute and relative effect sizes is recommended

Does your paper address CONSORT subitem 17b? *

Copy and paste relevant sections from the manuscript (include quotes in quotation marks "like this" to indicate direct quotes from your manuscript), or elaborate on this item by providing additional information not in the ms, or briefly explain why the item is not applicable/relevant for your study

Not applicable in the present study

18) Results of any other analyses performed, including subgroup analyses and adjusted analyses, distinguishing pre-specified from exploratory

Does your paper address CONSORT subitem 18? *

Copy and paste relevant sections from the manuscript (include quotes in quotation marks "like this" to indicate direct quotes from your manuscript), or elaborate on this item by providing additional information not in the ms, or briefly explain why the item is not applicable/relevant for your study

Not applicable in the present study

18-i) Subgroup analysis of comparing only users

A subgroup analysis of comparing only users is not uncommon in ehealth trials, but if done, it must be stressed that this is a self-selected sample and no longer an unbiased sample from a randomized trial (see 16-iii).

	1	2	3	4	5	
subitem not at all important	◉	○	○	○	○	essential

清除選取的項目

Does your paper address subitem 18-i?

Copy and paste relevant sections from the manuscript (include quotes in quotation marks "like this" to indicate direct quotes from your manuscript), or elaborate on this item by providing additional information not in the ms, or briefly explain why the item is not applicable/relevant for your study

Not applicable in the present study

19) All important harms or unintended effects in each group

(for specific guidance see CONSORT for harms)

Does your paper address CONSORT subitem 19? *

Copy and paste relevant sections from the manuscript (include quotes in quotation marks "like this" to indicate direct quotes from your manuscript), or elaborate on this item by providing additional information not in the ms, or briefly explain why the item is not applicable/relevant for your study

Not applicable in the present study

19-i) Include privacy breaches, technical problems

Include privacy breaches, technical problems. This does not only include physical “harm” to participants, but also incidents such as perceived or real privacy breaches [1], technical problems, and other unexpected/unintended incidents. “Unintended effects” also includes unintended positive effects [2].

	1	2	3	4	5	
subitem not at all important	◉	○	○	○	○	essential

清除選取的項目

Does your paper address subitem 19-i?

Copy and paste relevant sections from the manuscript (include quotes in quotation marks "like this" to indicate direct quotes from your manuscript), or elaborate on this item by providing additional information not in the ms, or briefly explain why the item is not applicable/relevant for your study

Not applicable in the present study

19-ii) Include qualitative feedback from participants or observations from staff/researchers

Include qualitative feedback from participants or observations from staff/researchers, if available, on strengths and shortcomings of the application, especially if they point to unintended/unexpected effects or uses. This includes (if available) reasons for why people did or did not use the application as intended by the developers.

	1	2	3	4	5	
subitem not at all important	◉	○	○	○	○	essential

清除選取的項目

Does your paper address subitem 19-ii?

Copy and paste relevant sections from the manuscript (include quotes in quotation marks "like this" to indicate direct quotes from your manuscript), or elaborate on this item by providing additional information not in the ms, or briefly explain why the item is not applicable/relevant for your study

A total of 76 healthcare providers (Supplementary Table S4 in Multimedia Appendix 3) responded to the survey request of clinical satisfaction of i-Dashboard (response rate 84.4%). The results regarding the perceived benefits of information management using the established EMR and implemented i-Dashboard during pre-rounding data collection and MDRs are shown in Table 4 and Supplementary Table S5 in Multimedia Appendix 3. For pre-rounding data gathering, timeliness, efficiency, clarity and presentation format were reported to be better in i-Dashboard than in the established EMR. Although no significant differences in data accuracy and information content sufficiency were reported between the established EMR and i-Dashboard, i-Dashboard made the task of data gathering significantly less difficult and mentally demanding. During MDRs, patient presentations using i-Dashboard were more accurate and organized, leading to a better understanding of the situation and goal. Moreover, compared to the established EMR, i-Dashboard enhanced communication, opinion exchange and team participation.
Finally, as shown in Table 5 and Supplementary Table S6 in Multimedia Appendix 3, the results regarding the intention to use and personal impact of i-Dashboard suggested that these participants were willing to use i-Dashboard continuously in association with the enhancement of situation awareness, care plan development and team participation whereas reduction of workload and complexity.

DISCUSSION

22) Interpretation consistent with results, balancing benefits and harms, and considering other relevant evidence

NPT: In addition, take into account the choice of the comparator, lack of or partial blinding, and unequal expertise of care providers or centers in each group

22-i) Restate study questions and summarize the answers suggested by the data, starting with primary outcomes and process outcomes (use)

Restate study questions and summarize the answers suggested by the data, starting with primary outcomes and process outcomes (use).

	1	2	3	4	5	
subitem not at all important	○	○	○	○	◉	essential

清除選取的項目

Does your paper address subitem 22-i? *

Copy and paste relevant sections from the manuscript (include quotes in quotation marks "like this" to indicate direct quotes from your manuscript), or elaborate on this item by providing additional information not in the ms, or briefly explain why the item is not applicable/relevant for your study

Principal Findings
The i-Dashboard was developed as a structured process-oriented information platform for MDRs, where the efficiency of data retrieval, fidelity of data communication and satisfaction of interdisciplinary communication are all highly required. Through a comprehensive evaluation of the performance of i-Dashboard, we found that under similar disease complexity, i-Dashboard may increase efficiency in pre-rounding data gathering compared to the established EMR. More important, displaying i-Dashboard on large touchscreens in MDRs may enhance communication accuracy, information exchange and clinical satisfaction.

22-ii) Highlight unanswered new questions, suggest future research

Highlight unanswered new questions, suggest future research.

	1	2	3	4	5	
subitem not at all important	○	○	○	○	◉	essential

清除選取的項目

Does your paper address subitem 22-ii?

Copy and paste relevant sections from the manuscript (include quotes in quotation marks "like this" to indicate direct quotes from your manuscript), or elaborate on this item by providing additional information not in the ms, or briefly explain why the item is not applicable/relevant for your study

The present findings must be interpreted within the context of the study limitations. First, traditional study outcomes in critical care, such as mortality and length of stay, were not evaluated in the present study. No conclusion can be achieved regarding the effect of i-Dashboard on patient outcomes. Further studies are warranted in this respect.

20) Trial limitations, addressing sources of potential bias, imprecision, and, if relevant, multiplicity of analyses

20-i) Typical limitations in ehealth trials

Typical limitations in ehealth trials: Participants in ehealth trials are rarely blinded. Ehealth trials often look at a multiplicity of outcomes, increasing risk for a Type I error. Discuss biases due to non-use of the intervention/usability issues, biases through informed consent procedures, unexpected events.

	1	2	3	4	5	
subitem not at all important	○	○	○	○	◉	essential

清除選取的項目

Does your paper address subitem 20-i? *

Copy and paste relevant sections from the manuscript (include quotes in quotation marks "like this" to indicate direct quotes from your manuscript), or elaborate on this item by providing additional information not in the ms, or briefly explain why the item is not applicable/relevant for your study

Second, the designers of i-Dashboard were part of the authors who conducted the study and assessed outcomes. Successful implementation of dashboards greatly relies on user experience. However, the participation of authors in the development process potentially leads to biases in assessing outcomes [26].

21) Generalisability (external validity, applicability) of the trial findings

NPT: External validity of the trial findings according to the intervention, comparators, patients, and care providers or centers involved in the trial

21-i) Generalizability to other populations

Generalizability to other populations: In particular, discuss generalizability to a general Internet population, outside of a RCT setting, and general patient population, including applicability of the study results for other organizations

	1	2	3	4	5	
subitem not at all important	○	○	○	○	◉	essential

清除選取的項目

Does your paper address subitem 21-i?

Copy and paste relevant sections from the manuscript (include quotes in quotation marks "like this" to indicate direct quotes from your manuscript), or elaborate on this item by providing additional information not in the ms, or briefly explain why the item is not applicable/relevant for your study

Finally, i-Dashboard was custom-developed with reference to our established EMR environment. Therefore, it is difficult to extrapolate our study findings directly into ICUs of other hospitals, possibly limiting the generalizability. Nevertheless, visualization dashboards are intended to reduce the time spent on the data gathering process and improve situation awareness and navigation [11, 21]. The promising results of i-Dashboard obtained in a mature MDR environment suggest that these design concepts may help develop or modify visualization dashboards in the ICU more applicable for MDRs through technological advancements.

21-ii) Discuss if there were elements in the RCT that would be different in a routine application setting

Discuss if there were elements in the RCT that would be different in a routine application setting (e.g., prompts/reminders, more human involvement, training sessions or other co-interventions) and what impact the omission of these elements could have on use, adoption, or outcomes if the intervention is applied outside of a RCT setting.

	1	2	3	4	5	
subitem not at all important	○	○	○	○	◉	essential

清除選取的項目

Does your paper address subitem 21-ii?

Copy and paste relevant sections from the manuscript (include quotes in quotation marks "like this" to indicate direct quotes from your manuscript), or elaborate on this item by providing additional information not in the ms, or briefly explain why the item is not applicable/relevant for your study

Comparison with Previous Work
Visualization dashboards can inform decision-making and support behavior change in public health and healthcare services [22-25]. A recent review of the literature suggests that the strength of evidence on the effect of ICU visualization dashboards remains low [9]. Of the four available randomized controlled trials, only Pickering et al. [12] found significant improvement compared to the preexisting EMR environment. Time spent for pre-rounding data gathering efforts is practical to evaluate information tools on MDRs [7]. In their study, participants who had access to the AWARE dashboard significantly decreased the data gathering time from 12 to 9 minutes per patient (P<.03). In the present study, we estimated a reduction of ~60 mouse clicks per patient using i-Dashboard versus the established EMR before the trial. For experienced NPs, this improvement can be translated into a 6-minute reduction, compatible with what has been observed (5.8 minutes) during the trial. Healthcare providers can reduce cognitive fatigue in the data extraction process and pay attention to more productive information.
The ICU is a dynamic world in which multiple information pathways and personnel interactions facilitate patient care. Specially-trained healthcare professionals in the ICU rely on interdisciplinary communication to make effective clinical decisions. Reliance on a single individual in patient data communication during MDRs represents a coping strategy for an EMR system that does not automatically provide an effective visualization display of the data needed [8]. Consistent with previous studies [8], we found that verbally shared communication of patient data during MDRs was prone to errors and inaccuracies, and most of the laboratory misrepresentations were omissions. Regardless of laboratory or non-laboratory data, these misrepresentations could be almost eliminated using i-Dashboard as a visualization aid for MDRs.
While structured presentation using checklists and explicit definitions of each healthcare provider's role may facilitate MDRs, allied healthcare provider perceptions of not being valued by rounding intensivists have been recognized as an unfavorable factor that impairs the productivity of MDRs . In MDRs with i-Dashboard, the participants of different professionals had a space and time interval of their own, thereby enhancing the sense of participation and collaboration. i-Dashboard may help the RT, pharmacist and dietitian focus on expressing their thought explicitly, as other participants could catch numerical information through the visualization aid. Their inputs were thus more likely to be valued and adopted by the multidisciplinary care team. The positive impacts of i-Dashboard on objective outcomes were corroborated by the increased perception of situation awareness and team participation in participants, as revealed in questionnaire results.
We found that after the study period, healthcare providers in study units started to propose novel ideas that might improve the usability of i-Dashboard (e.g., rearranging the layout and increasing laboratory items). A recent study has demonstrated valuable experience regarding the evolution process of an emergency department dashboard, which has undergone several major revisions to respond to feedback from users [21]. Currently, we are working out the second version of i-Dashboard.

OTHER INFORMATION

23) Registration number and name of trial registry

Does your paper address CONSORT subitem 23? *

Copy and paste relevant sections from the manuscript (include quotes in quotation marks "like this" to indicate direct quotes from your manuscript), or elaborate on this item by providing additional information not in the ms, or briefly explain why the item is not applicable/relevant for your study

ClinicalTrials.gov NCT04845698; i-Dashboard for Multi-disciplinary Rounds in SICU

24) Where the full trial protocol can be accessed, if available

Does your paper address CONSORT subitem 24? *

Cite a Multimedia Appendix, other reference, or copy and paste relevant sections from the manuscript (include quotes in quotation marks "like this" to indicate direct quotes from your manuscript), or elaborate on this item by providing additional information not in the ms, or briefly explain why the item is not applicable/relevant for your study

https://clinicaltrials.gov/ct2/show/NCT04845698

25) Sources of funding and other support (such as supply of drugs), role of funders

Does your paper address CONSORT subitem 25? *

Copy and paste relevant sections from the manuscript (include quotes in quotation marks "like this" to indicate direct quotes from your manuscript), or elaborate on this item by providing additional information not in the ms, or briefly explain why the item is not applicable/relevant for your study

This work is supported by grants from the Ministry of Science and Technology, Executive Yuan, Taiwan (MOST 109-2634-F-006-023 to M-RS). This funding source had no role in the design of this study and will not have any role during its execution, analyses, interpretation of the data, or decision to submit results.

X27) Conflicts of Interest (not a CONSORT item)

X27-i) State the relation of the study team towards the system being evaluated

In addition to the usual declaration of interests (financial or otherwise), also state the relation of the study team towards the system being evaluated, i.e., state if the authors/evaluators are distinct from or identical with the developers/sponsors of the intervention.

	1	2	3	4	5	
subitem not at all important	○	○	○	○	◉	essential

清除選取的項目

Does your paper address subitem X27-i?

Copy and paste relevant sections from the manuscript (include quotes in quotation marks "like this" to indicate direct quotes from your manuscript), or elaborate on this item by providing additional information not in the ms, or briefly explain why the item is not applicable/relevant for your study

All authors declare no potential conflicts of interest.

About the CONSORT EHEALTH checklist

As a result of using this checklist, did you make changes in your manuscript? *

- ○ yes, major changes
- ○ yes, minor changes
- ◉ no

What were the most important changes you made as a result of using this checklist?

您的回答

How much time did you spend on going through the checklist INCLUDING making changes in your manuscript *

approximately 40 minutes to go through the checklist

As a result of using this checklist, do you think your manuscript has improved? *

- (x) yes
- ( ) no
- ( ) 其他:

Would you like to become involved in the CONSORT EHEALTH group?

This would involve for example becoming involved in participating in a workshop and writing an "Explanation and Elaboration" document

- ( ) yes
- (x) no
- ( ) 其他:

清除選取的項目

Any other comments or questions on CONSORT EHEALTH

您的回答

STOP - Save this form as PDF before you click submit

To generate a record that you filled in this form, we recommend to generate a PDF of this page (on a Mac, simply select "print" and then select "print as PDF") before you submit it.

When you submit your (revised) paper to JMIR, please upload the PDF as supplementary file.

Don't worry if some text in the textboxes is cut off, as we still have the complete information in our database. Thank you!

Final step: Click submit !

Click submit so we have your answers in our database!

提交

清除表單

請勿利用 Google 表單送出密碼。

Google 並未認可或建立這項內容。 檢舉濫用情形 - 服務條款 - 隱私權政策

Google 表單
