# Supplementary material for: Integration of an Intensive Care Unit Visualization Dashboard (i-Dashboard) as a Platform to Facilitate Multidisciplinary Rounds: Cluster-Randomized Controlled Trial
Source: J Med Internet Res. 2022 May 13;24(5):e35981. doi: 10.2196/35981 (PMC9143774; doi:10.2196/35981)
Supplement: Multimedia Appendix 2 [file jmir_v24i5e35981_app2.pdf]

# *i*-Dashboard

Surgical ICU, Department of Surgery
National Cheng Kung University Hospital

# Architecture

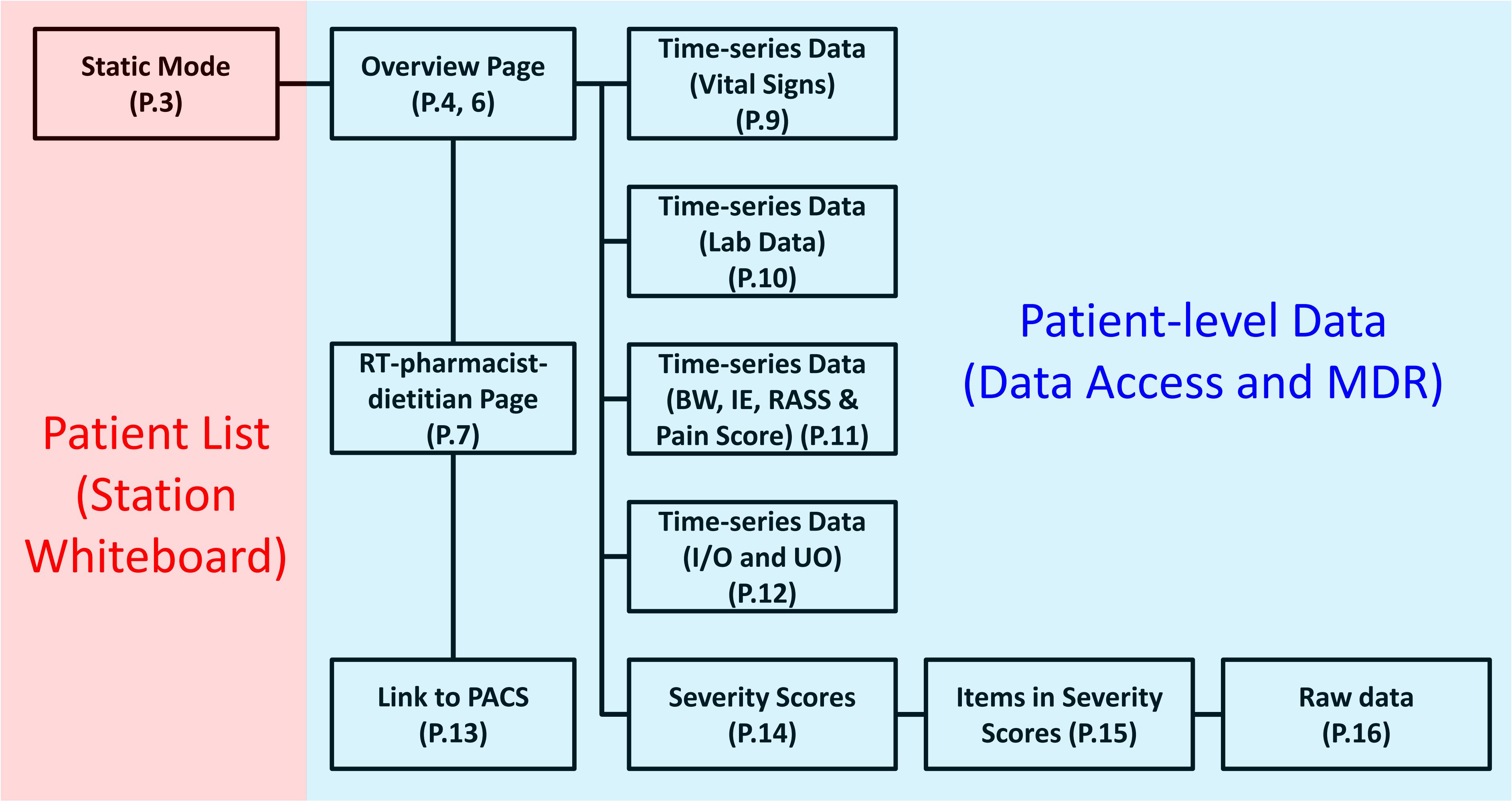

# Static Mode

## (Patient List and On-duty Healthcare Providers)

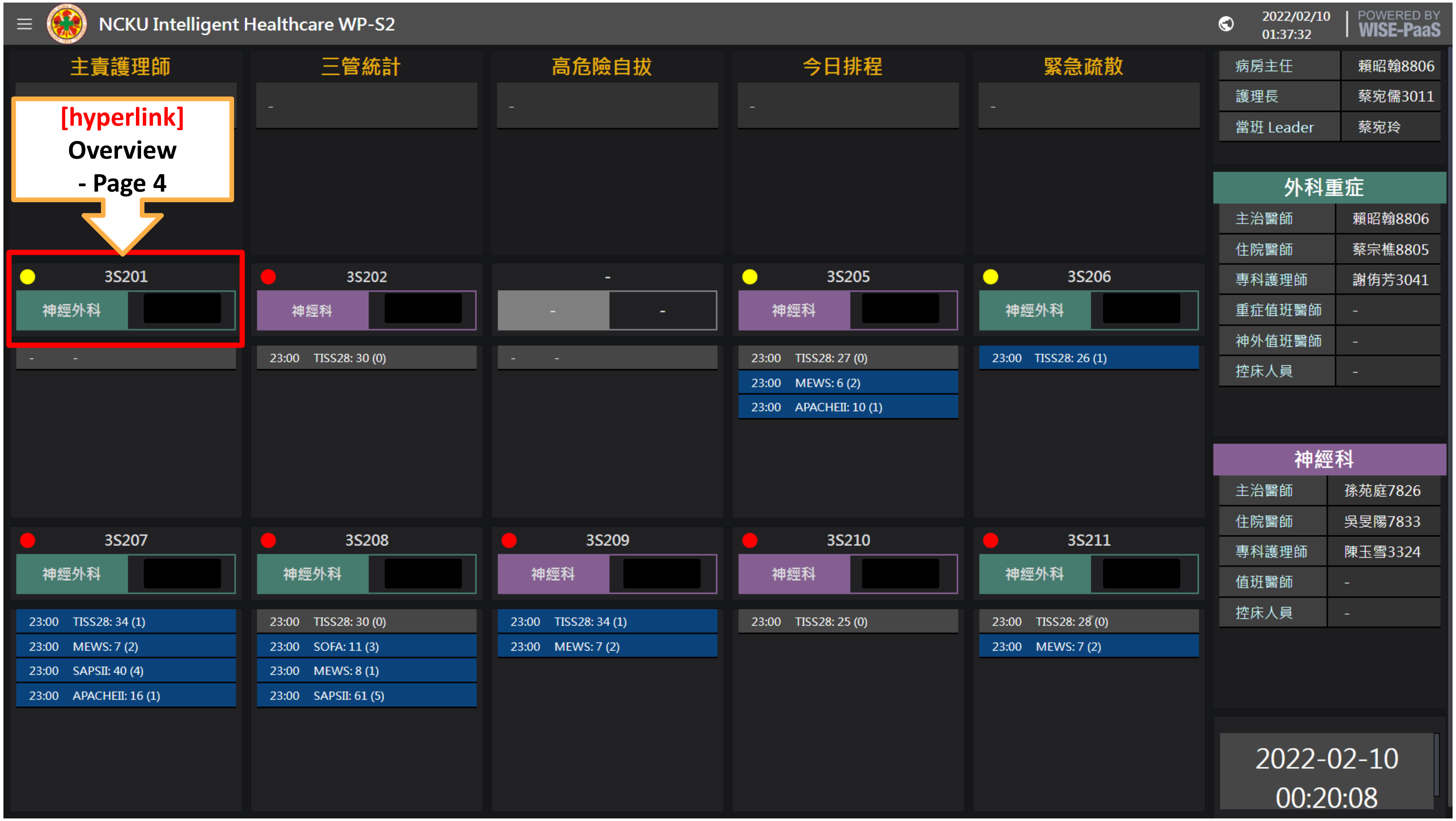

# Overview Page

[hyperlink]
Static Mode
- Page 3

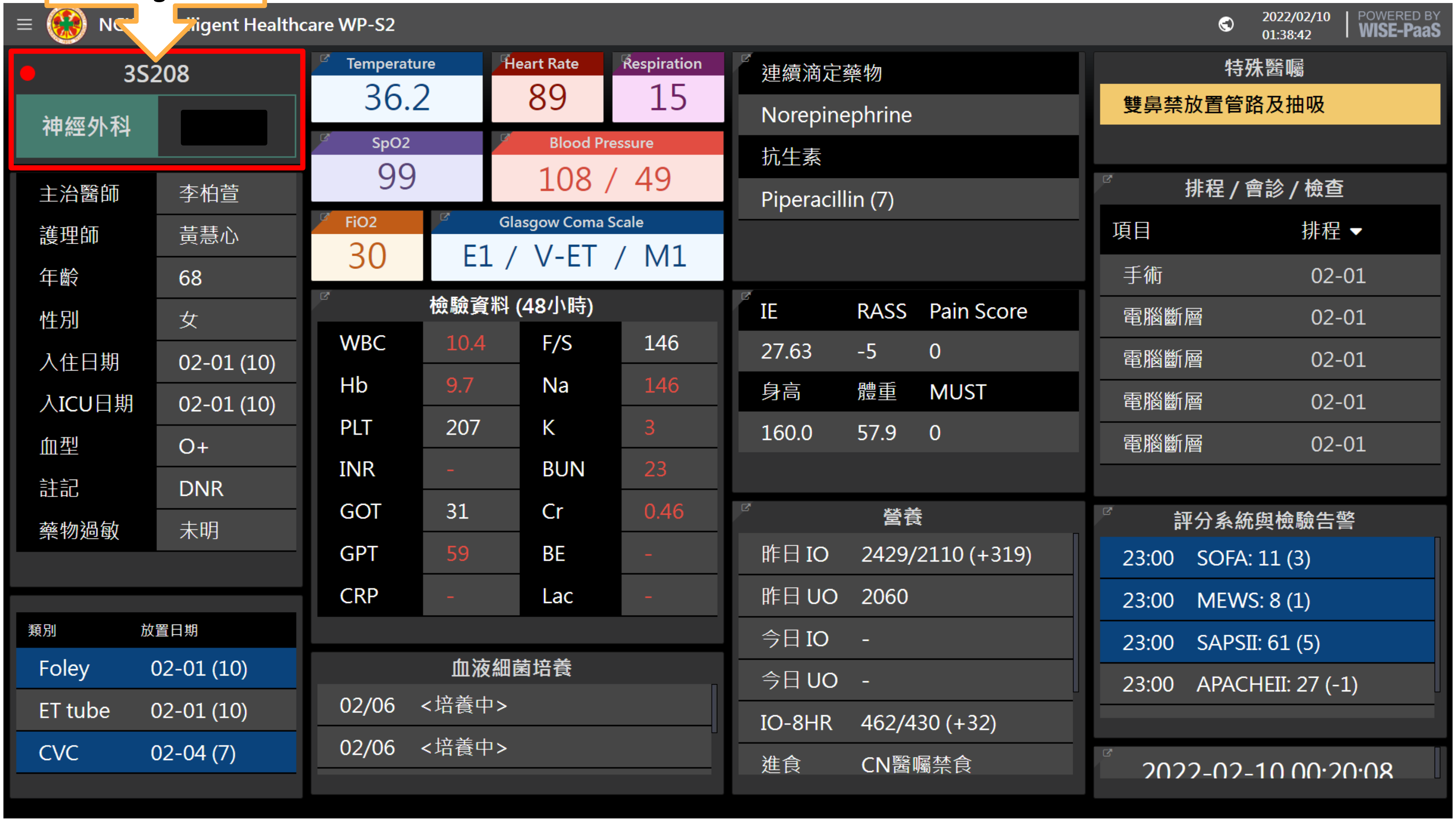

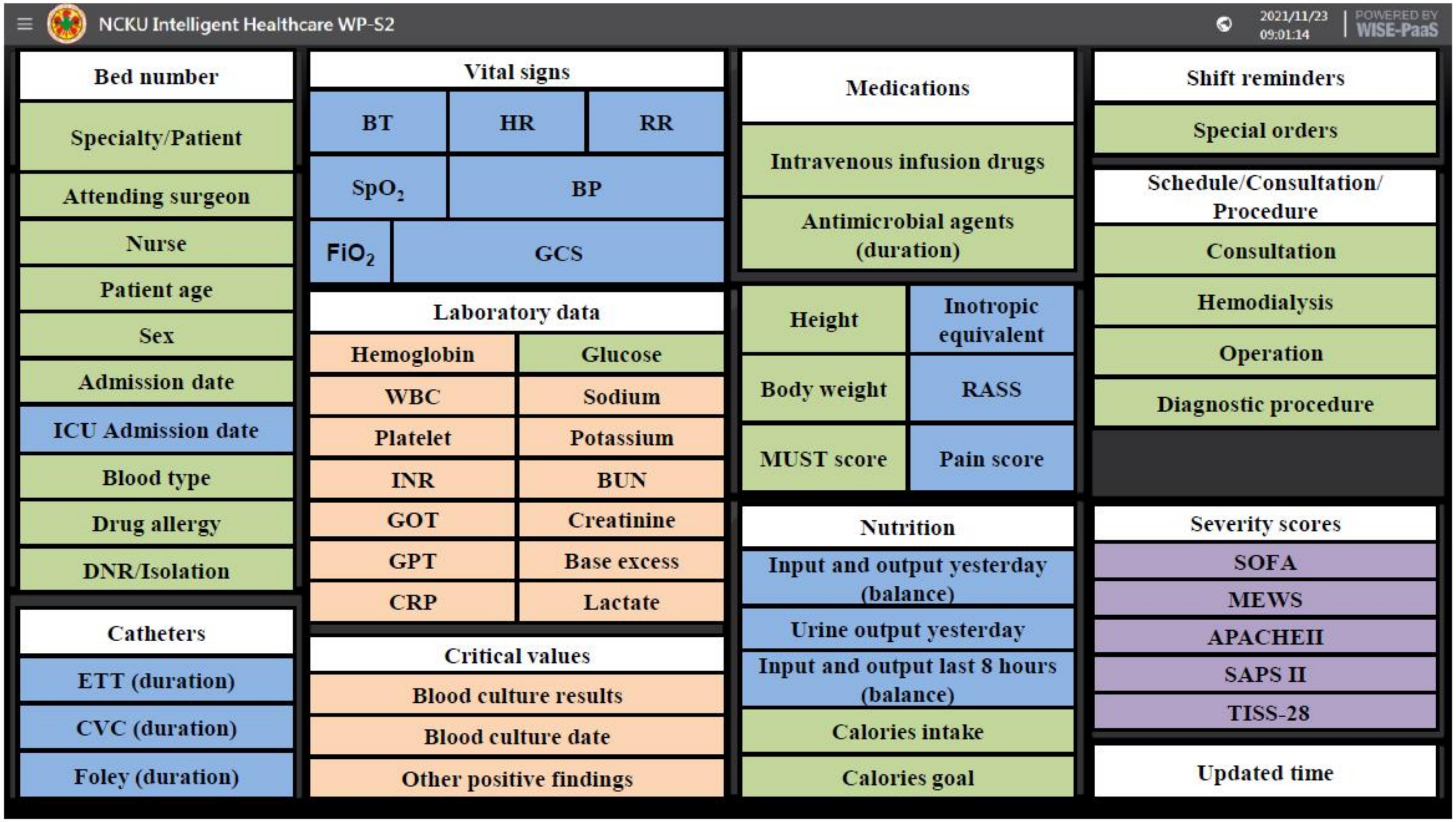


## Color Background and Data Origin

- HIS, Hospital Information System
- ICCA, Intellispace Critical Care and Anesthesia
- LIS, Laboratory Information System
- PACS, Picture Archiving and Communication System
- Built-in automated calculation

# Hyperlinks to Sublayers

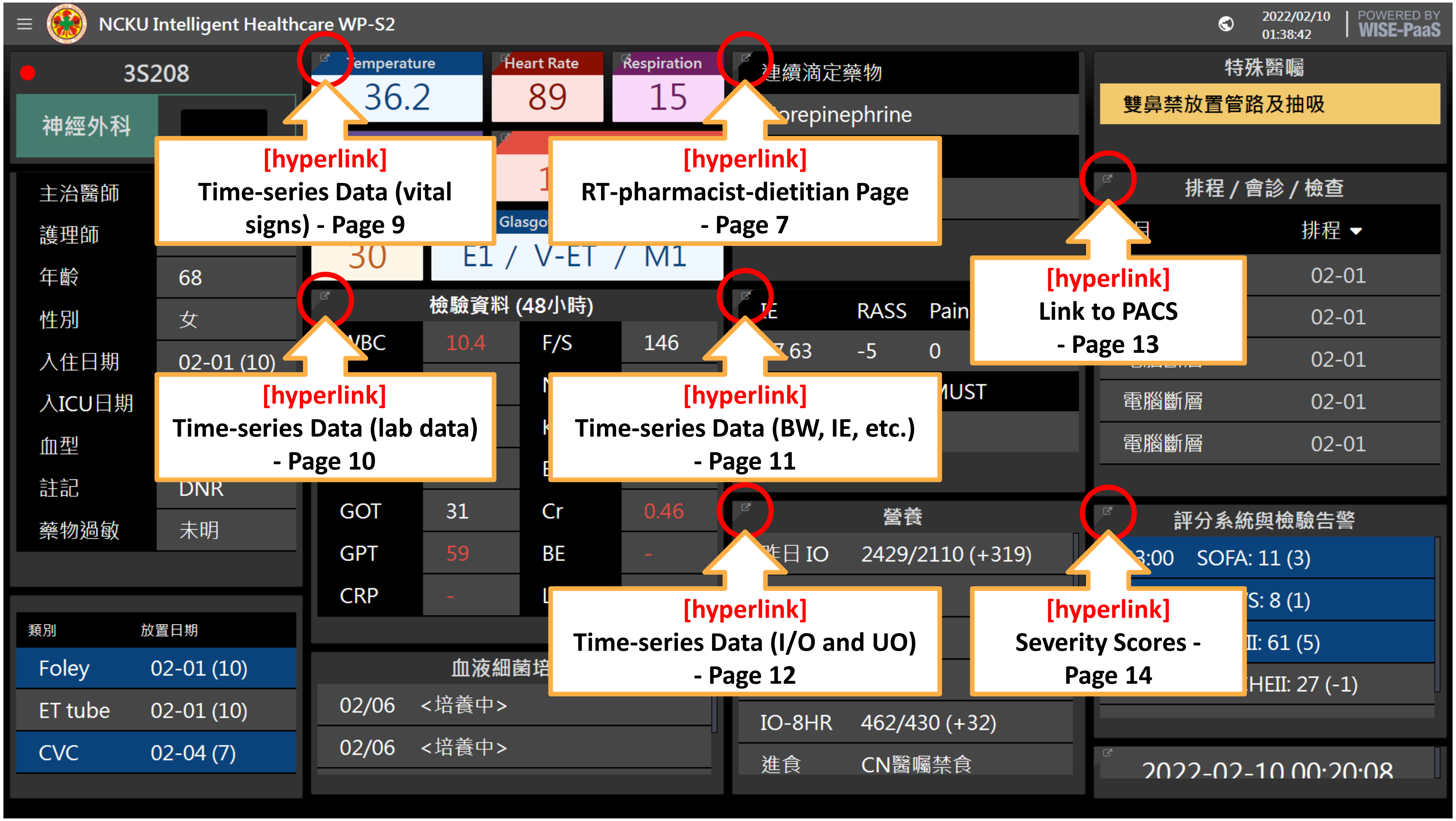

# RT-pharmacist-dietitian Page

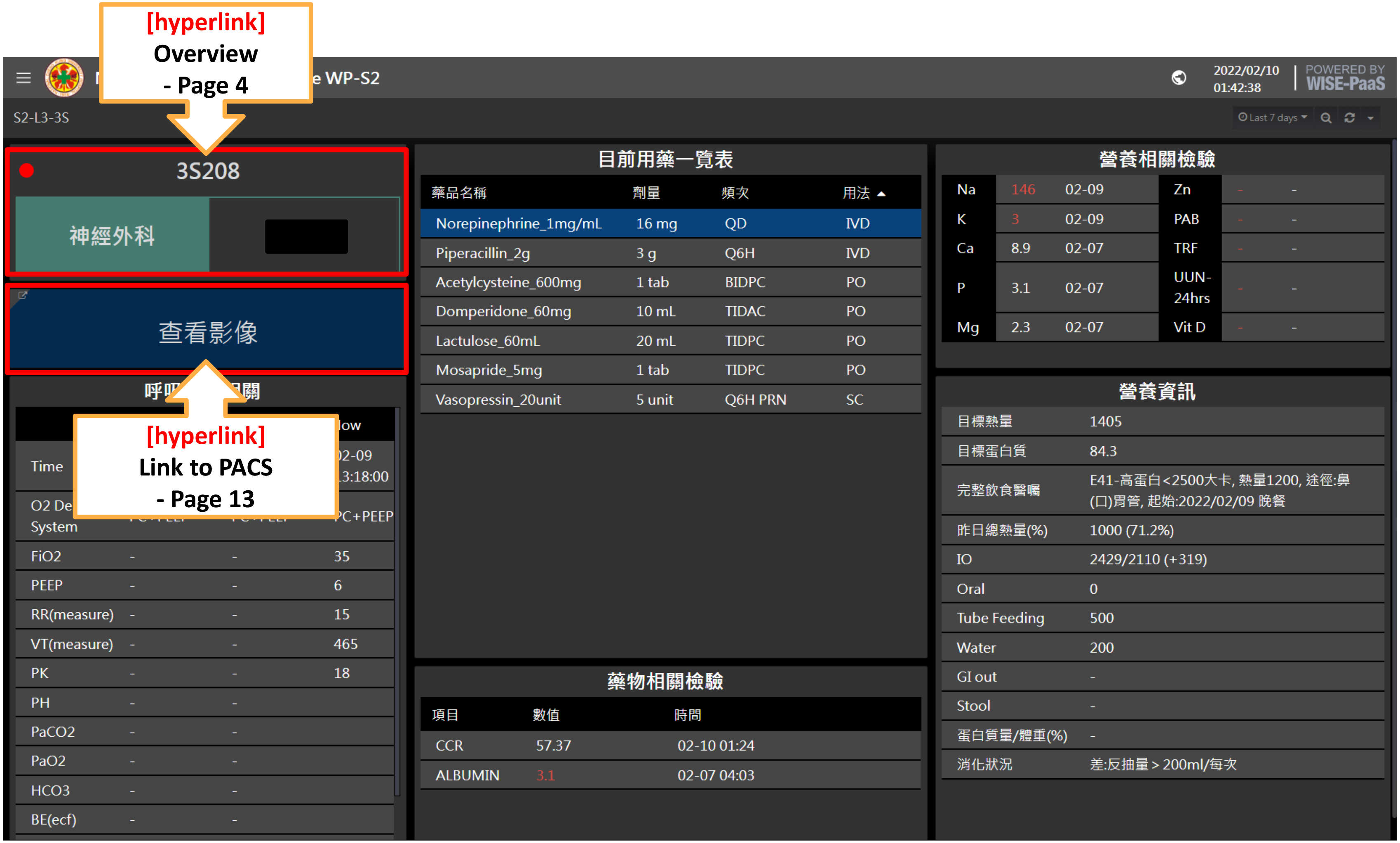

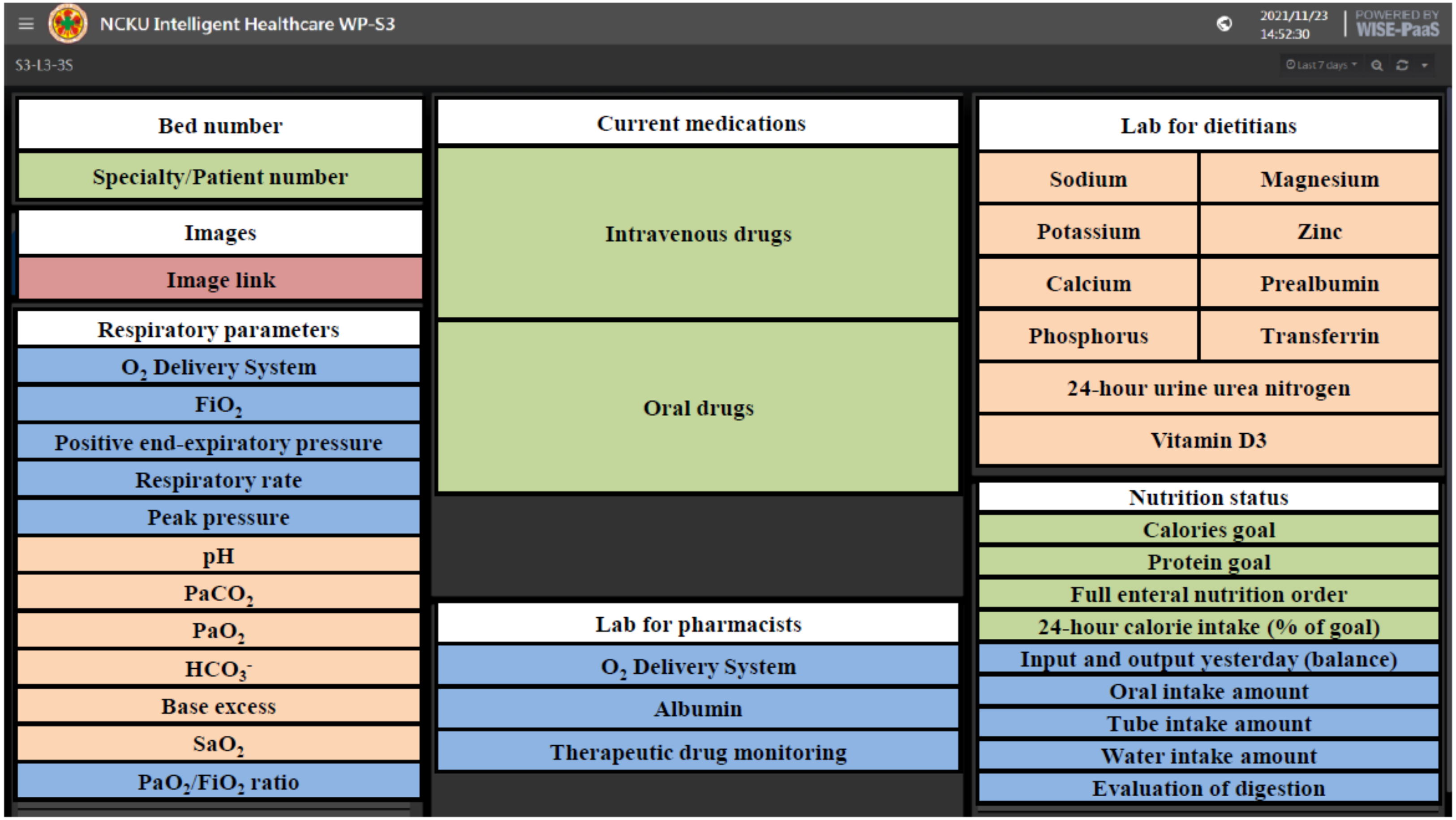


## Color Background and Data Origin

- HIS, Hospital Information System
- ICCA, Intellispace Critical Care and Anesthesia
- LIS, Laboratory Information System
- PACS, Picture Archiving and Communication System
- Built-in automated calculation

# Time-series Data (Vital Signs)

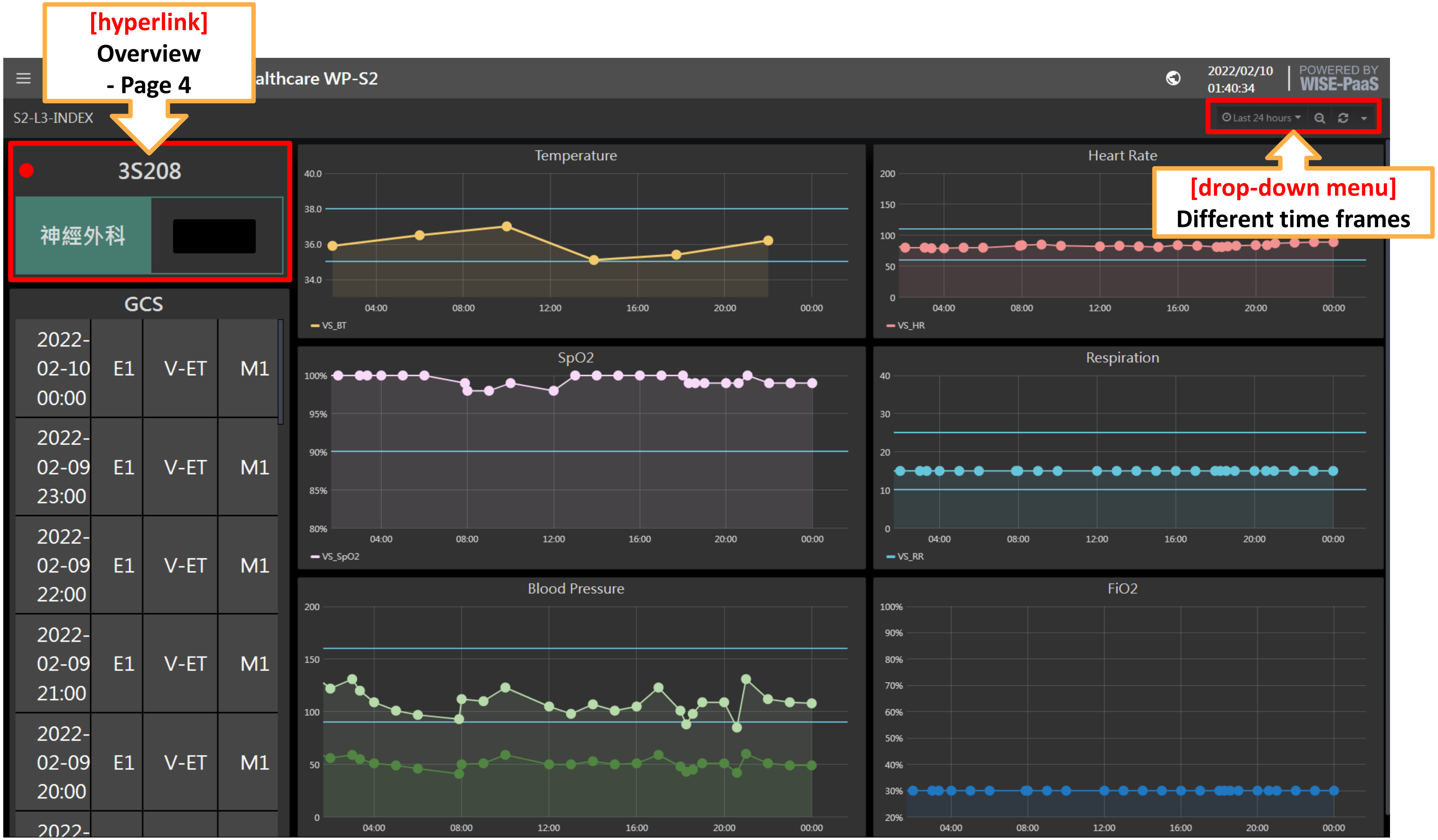

# Time-series Data (Lab Data)

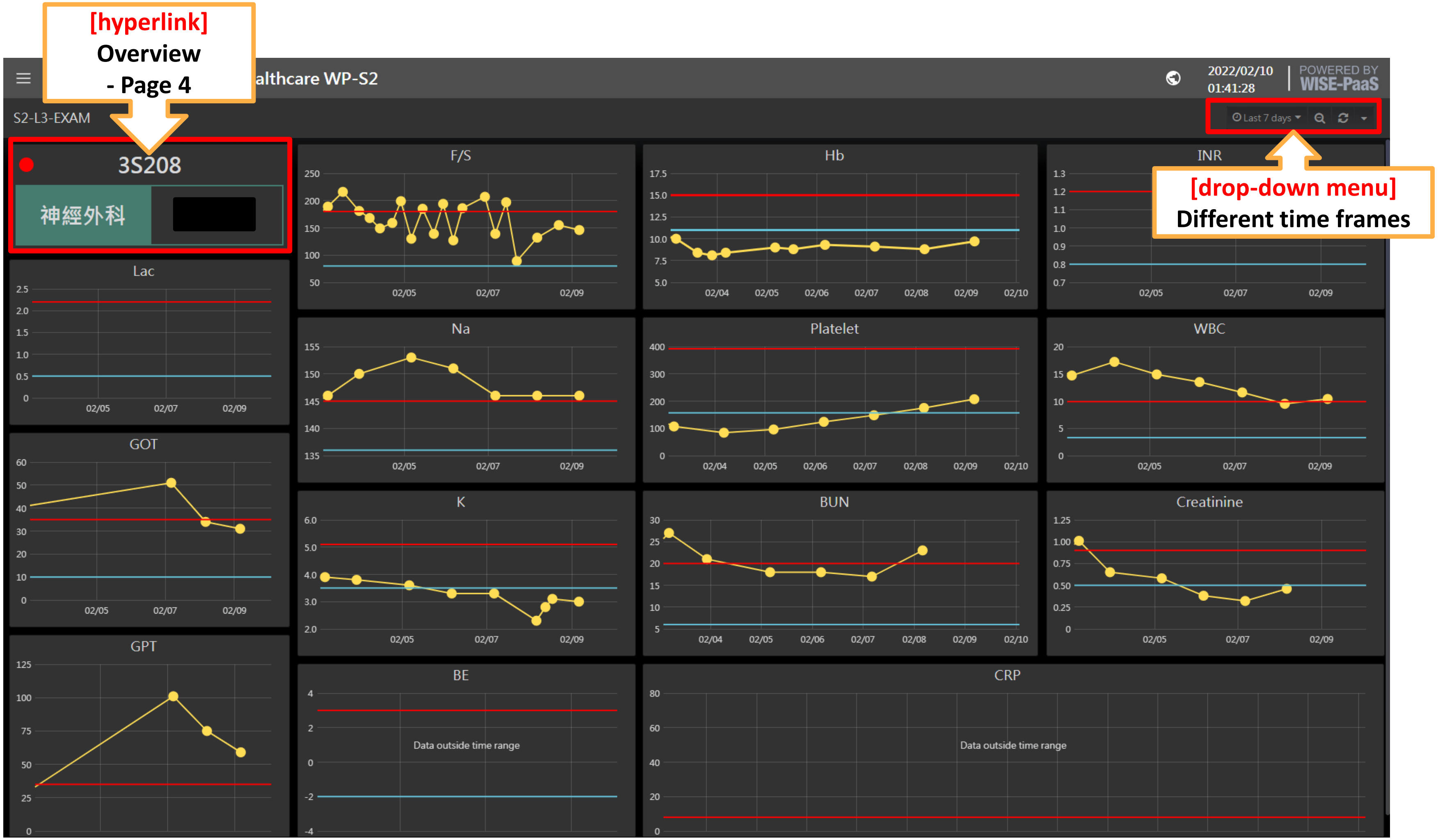

# Time-series Data
# (BW, IE, RASS & Pain Score)

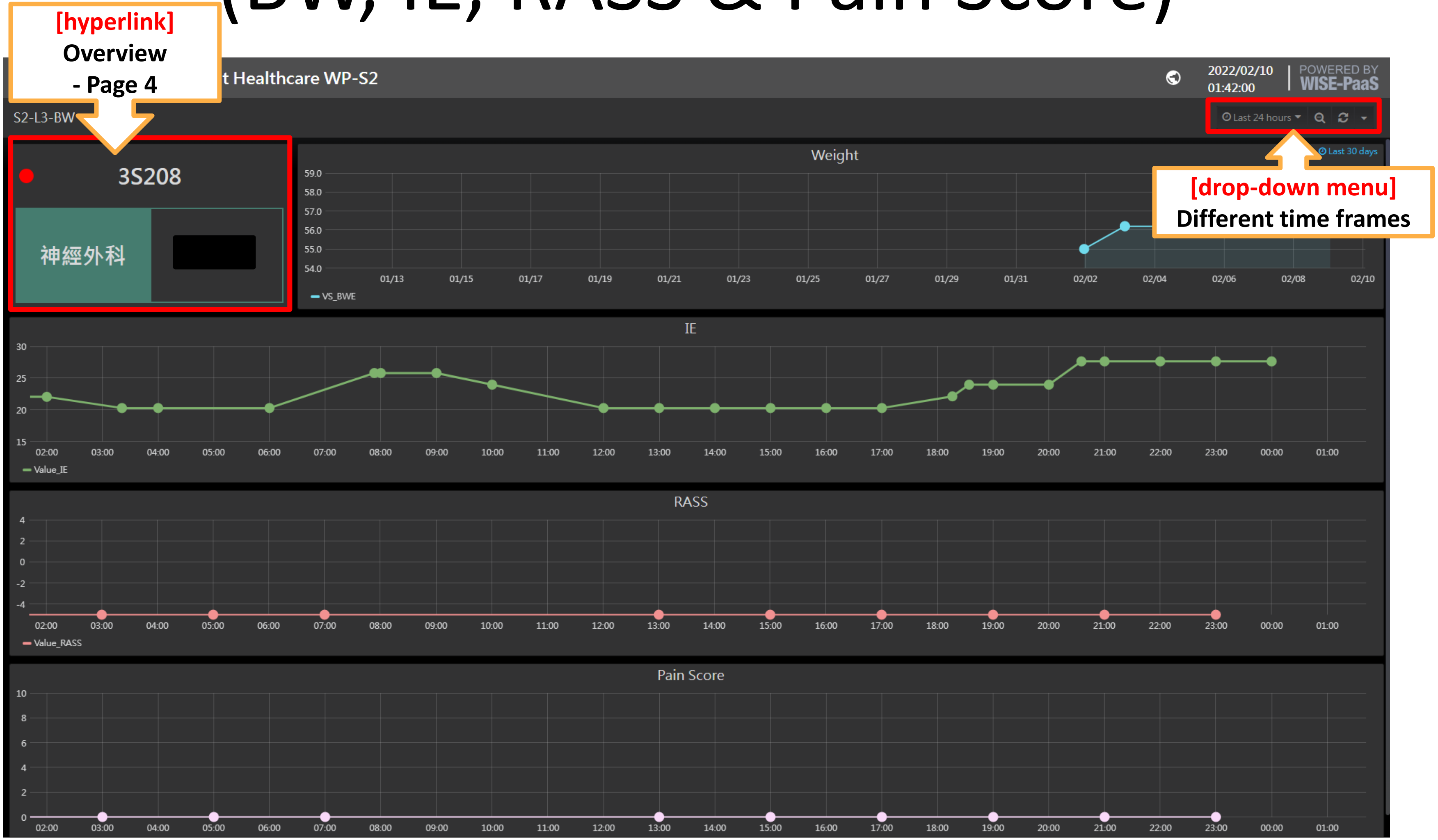

# Time-series Data (I/O and UO)

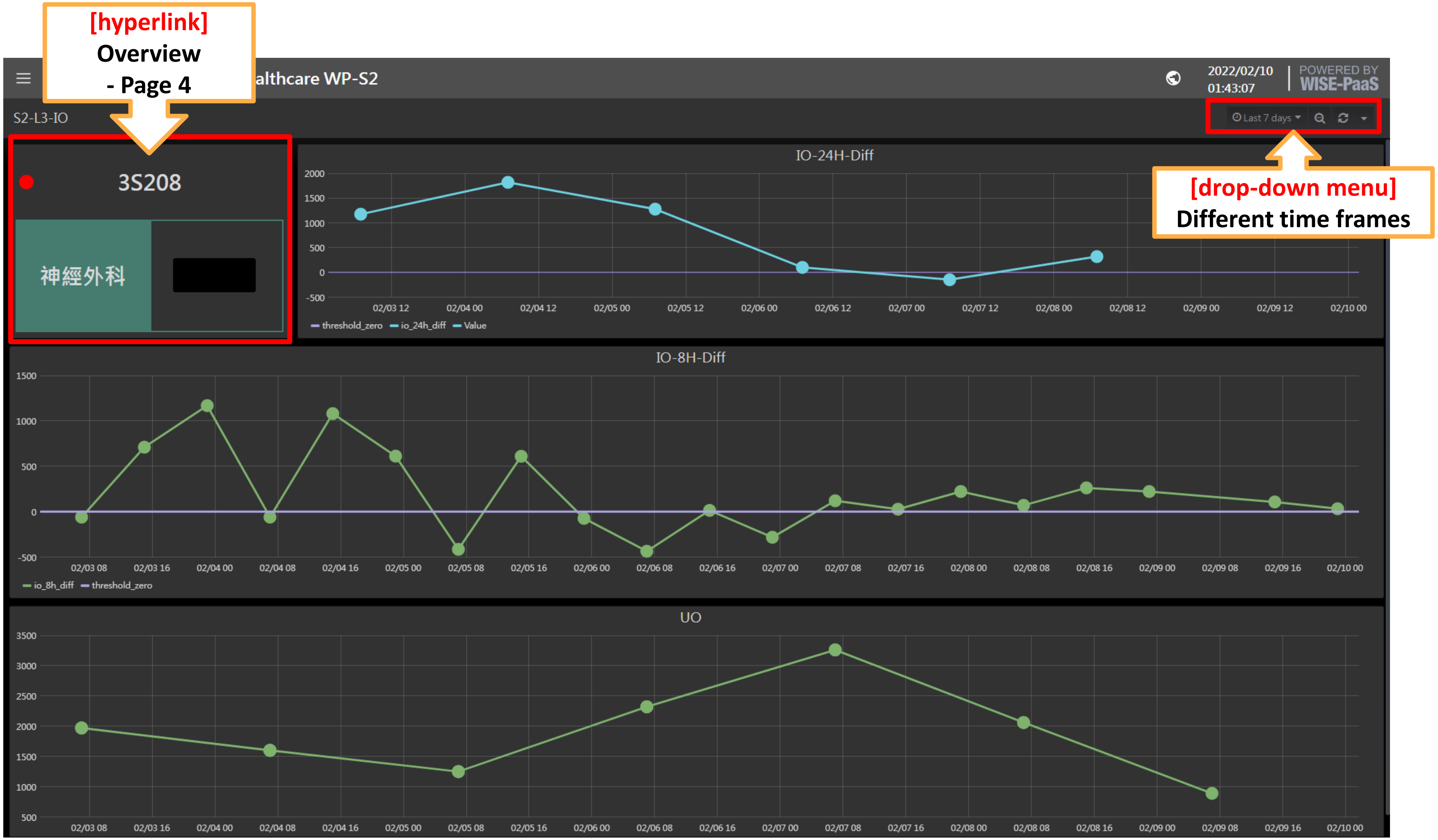

# Link to PACS

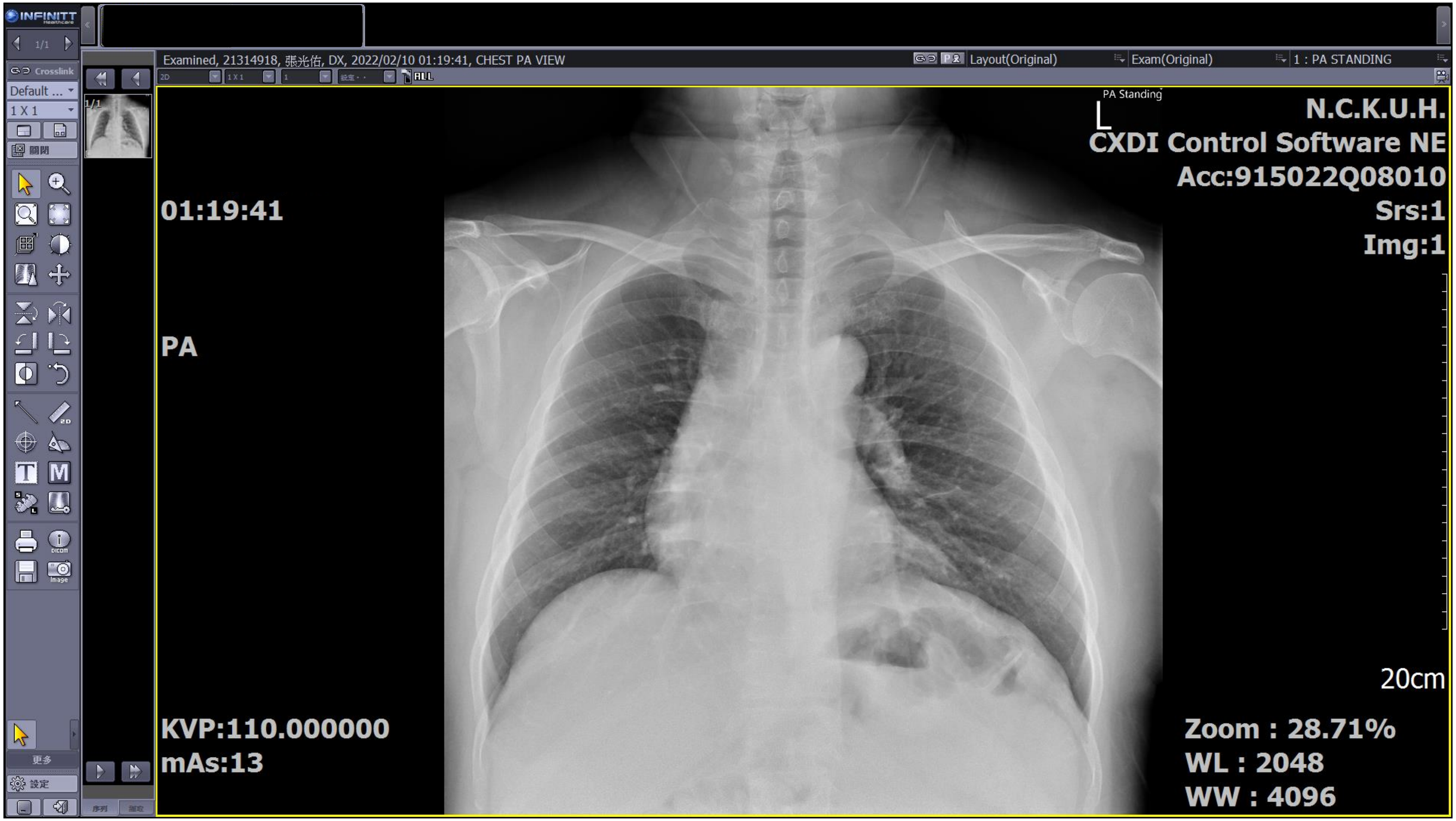
INFINITT
Crosslink
Default ...
1 X 1
Examined, 21314918, 張光佑, DX, 2022/02/10 01:19:41, CHEST PA VIEW
Layout(Original)
Exam(Original)
1 : PA STANDING
ALL
PA Standing
N.C.K.U.H.
CXDI Control Software NE
Acc:915022Q08010
Srs:1
Img:1
01:19:41
PA
20cm
KVP:110.000000
mAs:13
Zoom : 28.71%
WL : 2048
WW : 4096

# Severity Scores

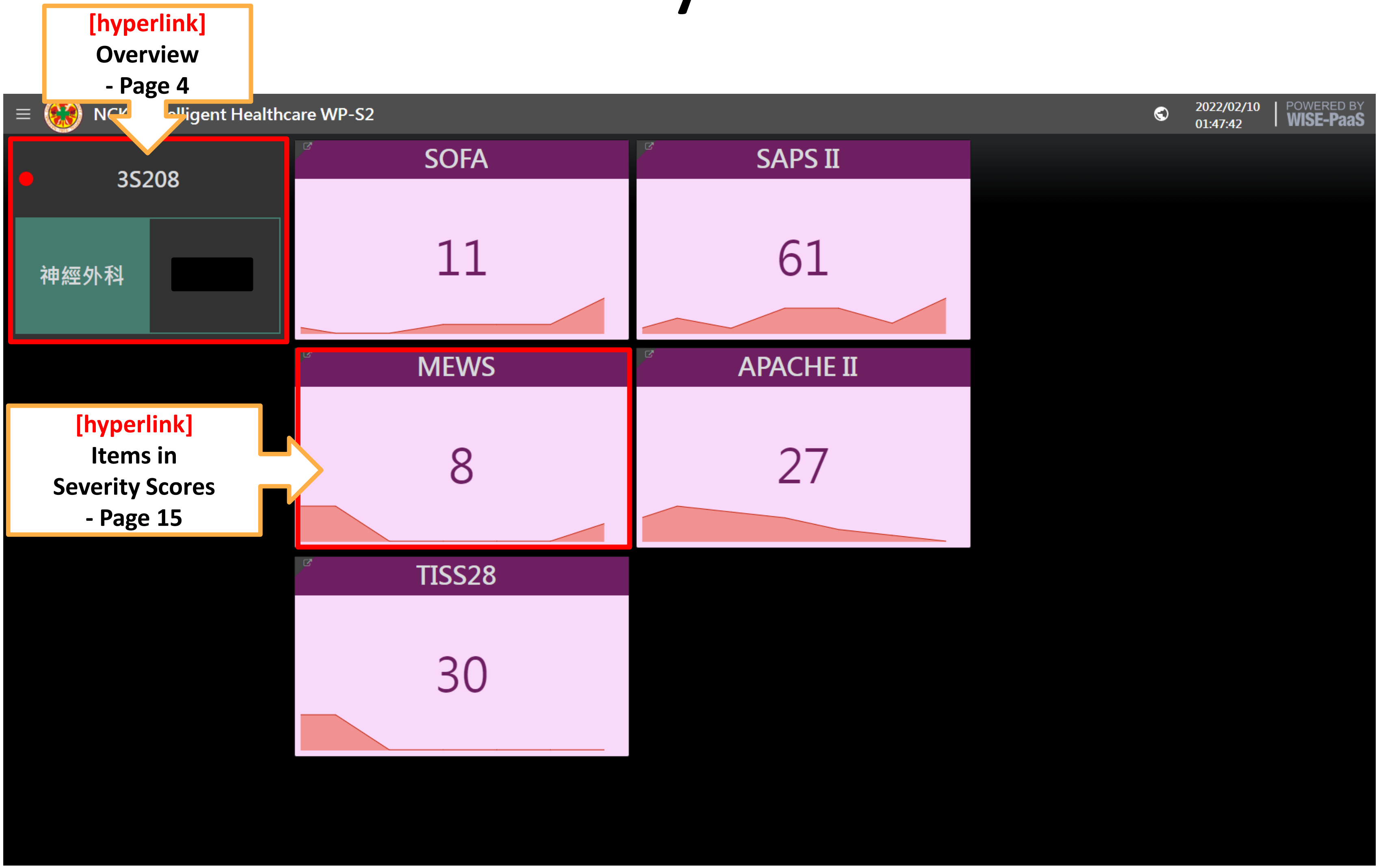

# Items in Severity Scores

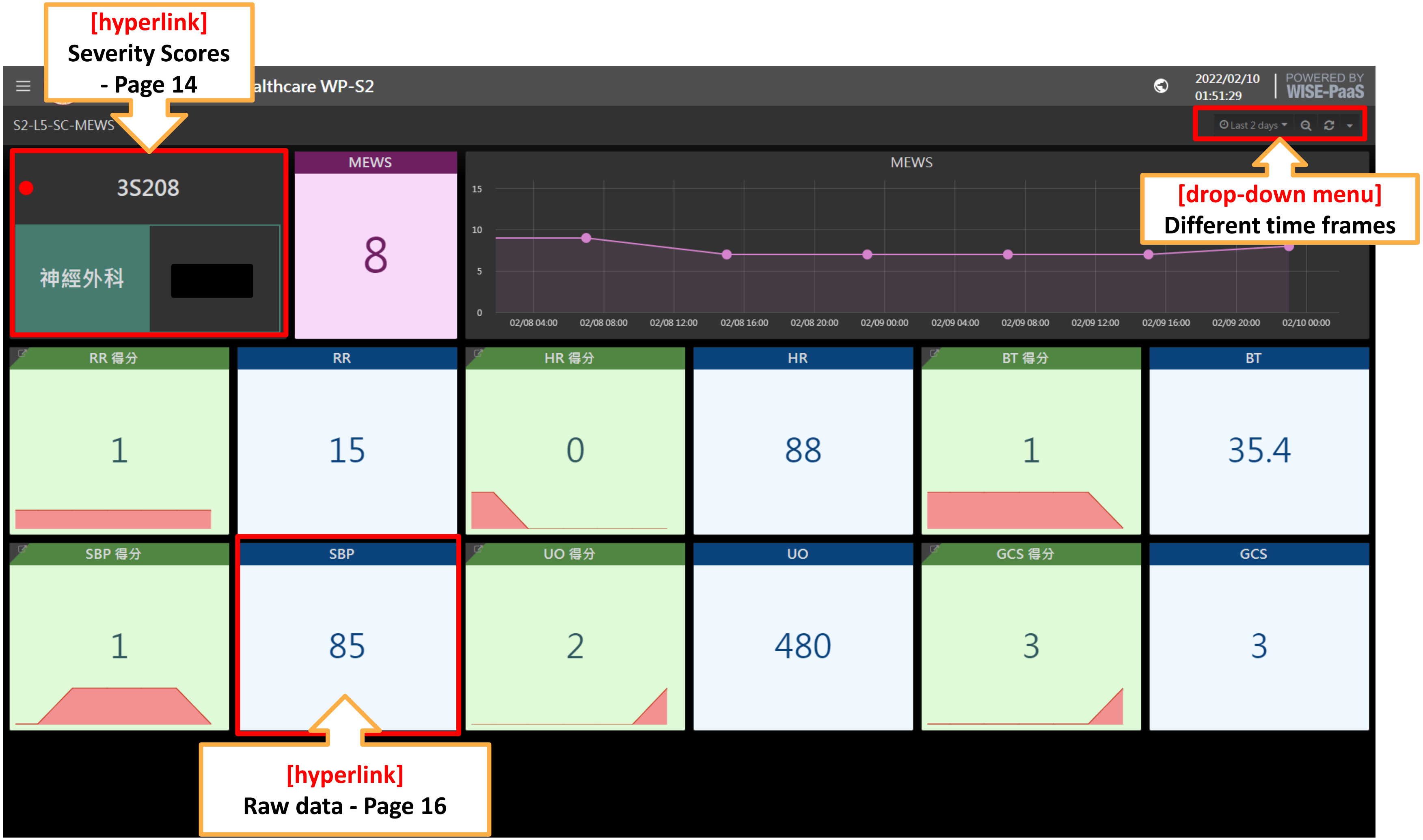

# Raw data

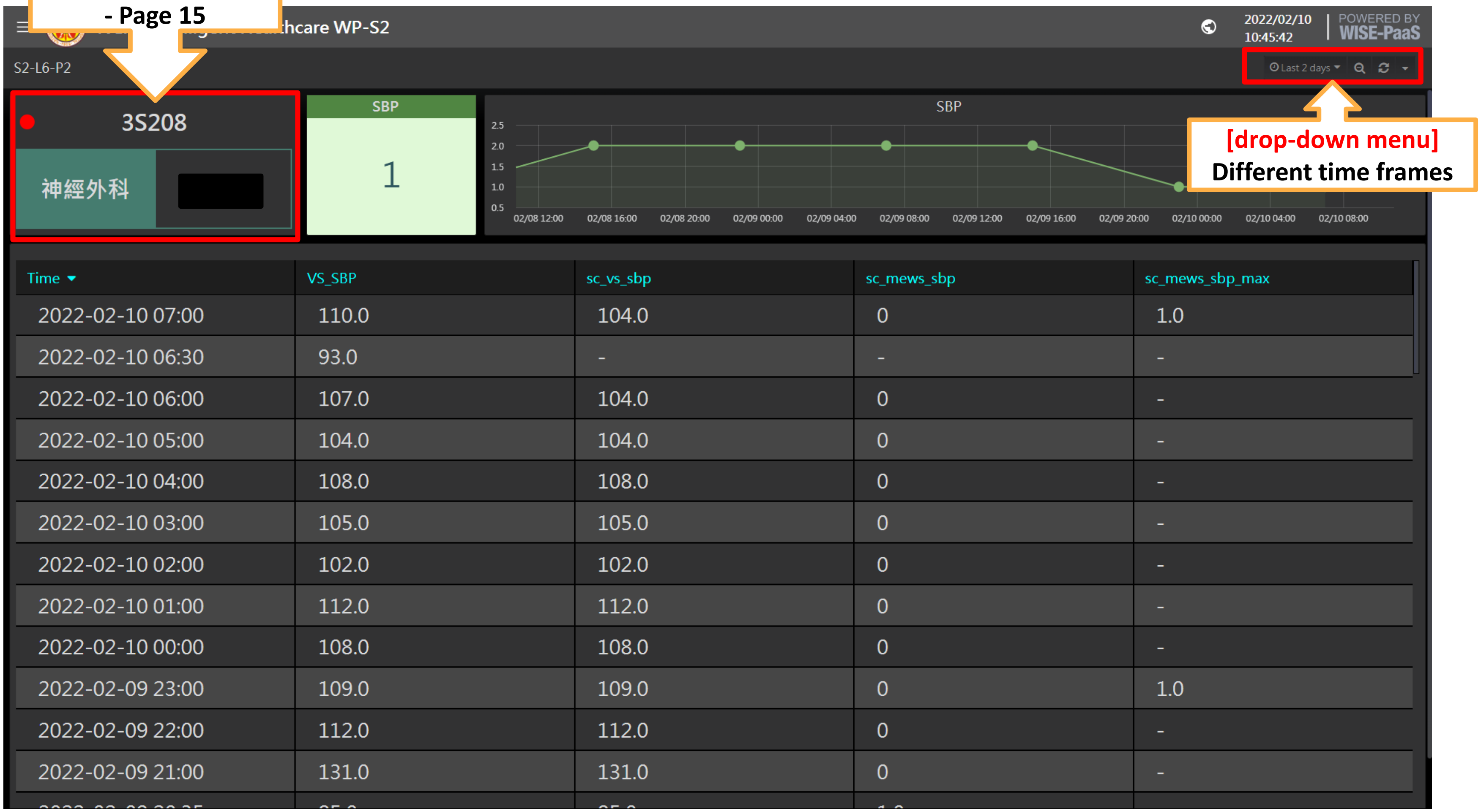


| Time | VS_SBP | sc_vs_sbp | sc_mews_sbp | sc_mews_sbp_max |
|---|---|---|---|---|
| 2022-02-10 07:00 | 110.0 | 104.0 | 0 | 1.0 |
| 2022-02-10 06:30 | 93.0 | - | - | - |
| 2022-02-10 06:00 | 107.0 | 104.0 | 0 | - |
| 2022-02-10 05:00 | 104.0 | 104.0 | 0 | - |
| 2022-02-10 04:00 | 108.0 | 108.0 | 0 | - |
| 2022-02-10 03:00 | 105.0 | 105.0 | 0 | - |
| 2022-02-10 02:00 | 102.0 | 102.0 | 0 | - |
| 2022-02-10 01:00 | 112.0 | 112.0 | 0 | - |
| 2022-02-10 00:00 | 108.0 | 108.0 | 0 | - |
| 2022-02-09 23:00 | 109.0 | 109.0 | 0 | 1.0 |
| 2022-02-09 22:00 | 112.0 | 112.0 | 0 | - |
| 2022-02-09 21:00 | 131.0 | 131.0 | 0 | - |
| [illegible] | [illegible] | [illegible] | [illegible] | |

# Alert

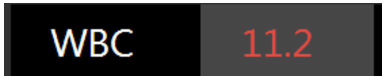


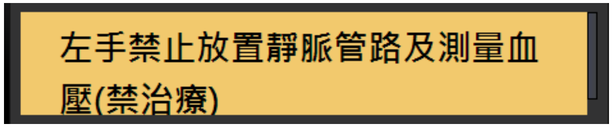


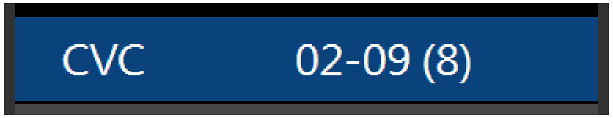


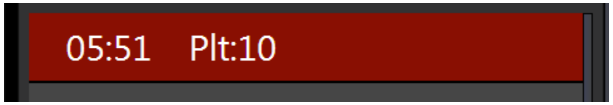


- The red text indicates abnormal values that are not critical but may require timely intervention (e.g., WBC 11.2K)
- The yellow background indicates special reminders (e.g., left arm venipuncture is prohibited)
- The blue background indicates clinical changes/deteriorations that potentially need attention (e.g., catheters used for 7 days or more)
- The red background indicates critical values that may require immediate clinical attention to avert significant patient morbidity or mortality (e.g., plt 10K)

# *Thank You
