# Supplementary material for: Integration of an Intensive Care Unit Visualization Dashboard (i-Dashboard) as a Platform to Facilitate Multidisciplinary Rounds: Cluster-Randomized Controlled Trial
Source: J Med Internet Res. 2022 May 13;24(5):e35981. doi: 10.2196/35981 (PMC9143774; doi:10.2196/35981)
Supplement: Multimedia Appendix 3 [file jmir_v24i5e35981_app3.pdf]

Multimedia Appendix 3. Structured script for data gathering and patient presentation.

Basic information<br>**Attending surgeon, patient age, admission date, drug allergy, DNR, isolation**
Catheter placement<br>**Presence of ETT, CVC, Foley catheter and their duration**
Vital signs over the past 24 hours<br>**BT:** exceeded the reference range (35~38°C)?<br>**HR:** exceeded the reference range (60~110/minute)? Arrhythmia?<br>**RR:** exceeded the reference range (10~25/minute)?<br>**SpO_2:** <90%?<br>**SBP:** exceeded the reference range (90~160 mmHg)?<br>**FiO_2:** oxygen demand increased?<br>**GCS:** any change?
Laboratory data over the past 24 hours **Abnormal values and trends (compared with previous data)?**<br>Hemogram/coagulation: **Hb/WBC/platelet/INR**<br>Electrolytes: **Na/K**<br>Renal function: **BUN/Cr**<br>Liver function: **GOT/GPT**<br>Others: **Glucose, lactate, BE, CRP**
Medications<br>**Intravenous infusion drugs?**<br>**Antimicrobial agents and duration?**<br>**Inotropic equivalent?**<br>**RASS?**<br>**Pain score?**
Input/output and nutrition<br>**Input and output yesterday?**<br>**Urine output yesterday?**<br>**MUST score?**<br>**Calories intake/goal?**
Critical values<br>**B/C results and date?**<br>**Other positive findings?**
Image studies over the past 24 hours<br>**Major findings?**
Consultations over the past 24 hours<br>**Which specialties?**
Other major events (e.g., operations, CPR) over the past 24 hours

DNR, do-not-resuscitate; ETT, endotracheal tube; CVC, central venous catheter; BT, body temperature; HR, heart rate; RR, respiration rate; SpO_2, oxygen saturation; SBP, systolic blood pressure; FiO_2, fraction of inspiration O_2; GCS, Glasgow coma scale; Hb, hemoglobin; WBC, white blood count; INR, international normalized ratio; Na, sodium; K, potassium; BUN, blood urea nitrogen; Cr, creatinine; GOT, glutamic oxaloacetic transaminase; GPT, glutamic pyruvic transaminase; BE, base excess; CRP, C-reactive protein; RASS, Richmond agitation-sedation scale; MUST, malnutrition universal screening tool; B/C, blood culture; CPR, cardiopulmonary resuscitation.
