# Supplementary material for: Integration of an Intensive Care Unit Visualization Dashboard (i-Dashboard) as a Platform to Facilitate Multidisciplinary Rounds: Cluster-Randomized Controlled Trial
Source: J Med Internet Res. 2022 May 13;24(5):e35981. doi: 10.2196/35981 (PMC9143774; doi:10.2196/35981)
Supplement: Multimedia Appendix 4 [file jmir_v24i5e35981_app4.pdf]

Multimedia Appendix 4. Standardized form for evaluating communication accuracy.

Date	Presenter	Observer
Patient number	Unit/Bed number	
Pre-rounding data gathering time: _____ minutes _____ seconds		

Non-laboratory data

		N/A	Correctly reported	Inaccurate			N/A	Correctly reported	Inaccurate
**Basic information**					**Medications**				
	Surgeon					IV infusion drug			
	Patient age					A/B (duration)			
	Admission date					IE			
	Drug allergy					RASS			
	DNR/isolation					Pain score			
**Catheters**					**Nutrition**				
	ETT (duration)					I/O			
	CVC (duration)					UO			
	Foley (duration)					MUST			
**Vital signs**						Calories intake			
	BT					Calories goal			
	HR				**Critical values**				
	RR					B/C results/date			
	SpO_2					Other findings			
	SBP				**Images**				
	FiO_2					Major findings			
	GCS				**Consultation**				

Laboratory data

	N/A	Correctly reported	Omission	Old data (newer results exist)	Pending result (result is already available)	Misinterpretation	Erroneous value
Hb							
WBC							
Platelet							
INR							
Na							
K							
BUN							
Cr							
GOT							
GPT							
Glucose							
Lactate							
BE							
CRP							

DNR, do-not-resuscitate; ETT, endotracheal tube, CVC, central venous catheter; BT, body temperature; HR, heart rate; RR, respiration rate; SpO_2, oxygen saturation; SBP, systolic blood pressure; FiO_2, fraction of inspiration O2; GCS, Glasgow coma scale; IV, intravenous; A/B, antibiotic (antimicrobial) agent; IE, inotropic equivalent; RASS, Richmond agitation-sedation scale; I/O, input and output, UO, urine output; MUST, malnutrition universal screening tool; B/C, blood culture; Hb, hemoglobin; WBC, white blood count; INR, international normalized ratio; Na, sodium; K, potassium; BUN, blood urea nitrogen; Cr, creatinine; GOT, glutamic oxaloacetic transaminase; GPT, glutamic pyruvic transaminase; BE, base excess; CRP, C-reactive protein.
