# Supplementary material for: Integration of an Intensive Care Unit Visualization Dashboard (i-Dashboard) as a Platform to Facilitate Multidisciplinary Rounds: Cluster-Randomized Controlled Trial
Source: J Med Internet Res. 2022 May 13;24(5):e35981. doi: 10.2196/35981 (PMC9143774; doi:10.2196/35981)
Supplement: Multimedia Appendix 5 [file jmir_v24i5e35981_app5.pdf]

Multimedia Appendix 5. Recommendations initiated by respiratory therapists, pharmacists and dietitians.

Professional/recommendation	Y or N
**Respiratory therapist**	
Weaning	
Liberation from mechanical ventilation	
Ventilator settings	
Medication- bronchodilators, acid-base balance regulating drugs, mucolytics	
Respiratory care- humidity therapy, vest therapy, muscle strengthening exercise	
Sedation break	
Tracheostomy	
**Pharmacist**	
Medication regimen modification	
Dosage/rate adjustment	
Route of administration	
Reminders of adverse drug reaction/drug-drug interaction	
Therapeutic drug monitoring	
Total parenteral nutrition-related issues	
**Dietitian**	
Formula Selection	
Calories requirement adjustment	
Measurement and monitoring (indirect calorimetry, urine urea nitrogen test, vitamin D3 and other laboratory data)	
Micronutrients supplements	
Electrolyte supplements	
Parenteral nutrition supplements	
Route of feeding	
Prokinetic drugs	
