# Supplementary material for: Integration of an Intensive Care Unit Visualization Dashboard (i-Dashboard) as a Platform to Facilitate Multidisciplinary Rounds: Cluster-Randomized Controlled Trial
Source: J Med Internet Res. 2022 May 13;24(5):e35981. doi: 10.2196/35981 (PMC9143774; doi:10.2196/35981)
Supplement: Multimedia Appendix 6 [file jmir_v24i5e35981_app6.pdf]

SURVEY QUESTIONNAIRE

(*i*-Dashboard for Multidisciplinary Rounds)

1. **Professional/Discipline**
   □ Physician
   □ Registered nurse
   □ Nurse practitioner
   □ Respiratory therapist
   □ Pharmacists
   □ Dietitians

2. **ICU experience**
   □ <1 year
   □ 1-3 years
   □ 3-5 years
   □ 5-10 years
   □ >10 years

3. **Questionnaire 1 (established EMR): Survey of perceived efficiency, accuracy and safety of information management using established EMR during pre-rounding data collection and MDRs**

**During pre-rounding data gathering**						
No.	Question	Strongly disagree	Disagree	Neutral	Agree	Strongly agree
Q1	Established EMR provides information catching up with condition changes.	1	2	3	4	5
Q2	Established EMR provides information that meets my demand for following MDRs.	1	2	3	4	5
Q3	Established EMR provides me sufficient information for patient care.	1	2	3	4	5
Q4	I get the information that I need in time using established EMR.	1	2	3	4	5
Q5	I get the information that I need using established EMR easily.	1	2	3	4	5
Q6	I am satisfied with the accuracy of the data using established EMR.	1	2	3	4	5
Q7	The information presented by established EMR is clear.	1	2	3	4	5
Q8	The information presented in the format of established EMR is effective and useful.	1	2	3	4	5
Q9	Established EMR makes data gathering difficult.	1	2	3	4	5
Q10	Data gathering with established EMR was a mentally demanding task.	1	2	3	4	5
**During MDRs**						
No.	Question	Strongly disagree	Disagree	Neutral	Agree	Strongly agree
Q11	The information presented using established EMR during MDRs was accurate.	1	2	3	4	5
Q12	The presentation of patient information during MDRs using established EMR was organized.	1	2	3	4	5
Q13	Established EMR makes me fully understanding the situation and goal of each patient.	1	2	3	4	5
Q14	Communication and opinion exchange in MDRs is enhanced using established EMR.	1	2	3	4	5
Q15	Developing care plans relies on joint decisions by team members using established EMR.	1	2	3	4	5

4. **Questionnaire 1 (*i*-Dashboard): Survey of perceived efficiency, accuracy and safety of information management using *i*-Dashboard during pre-rounding data collection and MDRs**

**During pre-rounding data gathering**						
No.	Question	Strongly disagree	Disagree	Neutral	Agree	Strongly agree
Q1	*i*-Dashboard provides information catching up with condition changes.	1	2	3	4	5
Q2	*i*-Dashboard provides information that meets my demand for following MDRs.	1	2	3	4	5
Q3	*i*-Dashboard provides me sufficient information for patient care.	1	2	3	4	5
Q4	I get the information that I need in time using *i*-Dashboard.	1	2	3	4	5
Q5	I get the information that I need using *i*-Dashboard easily.	1	2	3	4	5
Q6	I am satisfied with the accuracy of the data using *i*-Dashboard.	1	2	3	4	5
Q7	The information presented by *i*-Dashboard is clear.	1	2	3	4	5
Q8	The information presented in the format of *i*-Dashboard is effective and useful.	1	2	3	4	5
Q9	*i*-Dashboard makes data gathering difficult.	1	2	3	4	5
Q10	Data gathering with *i*-Dashboard was a mentally demanding task.	1	2	3	4	5
**During MDRs**						
No.	Question	Strongly disagree	Disagree	Neutral	Agree	Strongly agree
Q11	The information presented using *i*-Dashboard during MDRs was accurate.	1	2	3	4	5
Q12	The presentation of patient information during MDRs using *i*-Dashboard was organized.	1	2	3	4	5
Q13	*i*-Dashboard makes me fully understanding the situation and goal of each patient.	1	2	3	4	5
Q14	Communication and opinion exchange in MDRs is enhanced using *i*-Dashboard.	1	2	3	4	5
Q15	Developing care plans relies on joint decisions by team members using *i*-Dashboard.	1	2	3	4	5

5. Questionnaire 2: Survey of intention to use and personal impact of *i*-Dashboard on healthcare providers

No.	Question	Strongly disagree	Disagree	Neutral	Agree	Strongly agree
Q1	*i*-Dashboard is my primary tool to gather data on patients.	1	2	3	4	5
Q2	*i*-Dashboard is a tool that I am willing to use continuously.	1	2	3	4	5
Q3	*i*-Dashboard reduces time to recognize patient worsening and improving.	1	2	3	4	5
Q4	*i*-Dashboard increases the accuracy during data collection.	1	2	3	4	5
Q5	*i*-Dashboard reduces my workload.	1	2	3	4	5
Q6	*i*-Dashboard guides me in developing plans of care.	1	2	3	4	5
Q7	*i*-Dashboard makes me more effective as a team member.	1	2	3	4	5
Q8	*i*-Dashboard decreases the complexity of my job.	1	2	3	4	5

～～Thank You～～
