# Supplementary material for: Integration of an Intensive Care Unit Visualization Dashboard (i-Dashboard) as a Platform to Facilitate Multidisciplinary Rounds: Cluster-Randomized Controlled Trial
Source: J Med Internet Res. 2022 May 13;24(5):e35981. doi: 10.2196/35981 (PMC9143774; doi:10.2196/35981)
Supplement: Multimedia Appendix 7 [file jmir_v24i5e35981_app7.pdf]

**Multimedia Appendix 7. Questionnaire results**

**Table S1.** Characteristics of the 76 survey responders.

| Variable | Value |
|---|---|
| **Professional/discipline, n (%)** | |
| Physician | 9 (11.8%) |
| Nurse practitioner | 3 (3.9%) |
| Nurse | 43 (56.6%) |
| Respiratory therapist | 17 (22.4%) |
| Pharmacist | 2 (2.6%) |
| Nutritionist | 2 (2.6%) |
| **ICU experience (year), n (%)** | |
| <1 | 3 (3.9%) |
| 1-2 | 18 (23.7%) |
| 3-4 | 15 (19.7%) |
| 5-9 | 23 (30.3%) |
| >10 | 17 (22.4%) |

ICU, intensive care unit.

**Table S2.** Details of responses to Questionnaire 1 (n=76).

| Question | Environment | Response, n (%) | | | | | Points, mean (SD) |
|---|---|---|---|---|---|---|---|
| | | Strongly disagree | Disagree | Neutral | Agree | Strongly agree | |
| **During pre-rounding data gathering** | | | | | | | |
| Q1 | Established EMR | 0 (0) | 5 (6.6%) | 24 (31.6%) | 37 (48.7%) | 10 (13.2%) | 3.68 (0.79) |
| | *i*-Dashboard | 0 (0) | 1 (1.3%) | 10 (13.2%) | 40 (52.6%) | 25 (32.9%) | 4.17 (0.70) |
| Q2 | Established EMR | 0 (0) | 0 (0) | 11 (14.5%) | 45 (59.2%) | 20 (26.3%) | 4.12 (0.63) |
| | *i*-Dashboard | 0 (0) | 0 (0) | 6 (7.9%) | 42 (55.3%) | 28 (36.8%) | 4.29 (0.61) |
| Q3 | Established EMR | 0 (0) | 1 (1.3%) | 16 (21.1%) | 41 (53.9%) | 18 (23.7%) | 4.00 (0.71) |
| | *i*-Dashboard | 0 (0) | 1 (1.3%) | 7 (9.2%) | 47 (61.8%) | 21 (27.6%) | 4.16 (0.63) |
| Q4 | Established EMR | 0 (0) | 2 (2.6%) | 20 (26.3%) | 44 (57.9%) | 10 (13.2%) | 3.82 (0.69) |
| | *i*-Dashboard | 0 (0) | 1 (1.3%) | 6 (7.9%) | 52 (68.4%) | 17 (22.4%) | 4.12 (0.59) |
| Q5 | Established EMR | 0 (0) | 6 (7.9%) | 27 (35.5%) | 34 (44.7%) | 9 (11.8%) | 3.61 (0.80) |
| | *i*-Dashboard | 0 (0) | 0 (0) | 12 (15.8%) | 37 (48.7%) | 27 (35.5%) | 4.20 (0.69) |
| Q6 | Established EMR | 0 (0) | 1 (1.3%) | 20 (26.3%) | 43 (56.6%) | 12 (15.8%) | 3.87 (0.68) |
| | *i*-Dashboard | 0 (0) | 0 (0) | 14 (18.4%) | 46 (60.5%) | 16 (21.1%) | 4.03 (0.63) |
| Q7 | Established EMR | 0 (0) | 2 (2.6%) | 23 (30.3%) | 41 (53.9%) | 10 (13.2%) | 3.78 (0.70) |
| | *i*-Dashboard | 0 (0) | 0 (0) | 7 (9.2%) | 43 (56.6%) | 26 (34.2%) | 4.25 (0.61) |
| Q8 | Established EMR | 0 (0) | 4 (5.3%) | 26 (34.2%) | 37 (48.7%) | 9 (11.8%) | 3.67 (0.76) |
| | *i*-Dashboard | 0 (0) | 0 (0) | 7 (9.2%) | 40 (52.6%) | 29 (38.2%) | 4.29 (0.63) |
| Q9 | Established EMR | 1 (1.3%) | 28 (36.8%) | 23 (30.3%) | 21 (27.6%) | 3 (3.9%) | 2.96 (0.93) |
| | *i*-Dashboard | 10 (13.2%) | 31 (40.8%) | 18 (23.7%) | 15 (19.7%) | 2 (2.6%) | 2.58 (1.04) |
| Q10 | Established EMR | 1 (1.3%) | 24 (31.6%) | 25 (32.9%) | 19 (25.0%) | 7 (9.2%) | 3.09 (1.00) |
| | *i*-Dashboard | 15 (19.7%) | 30 (39.5%) | 18 (23.7%) | 10 (13.2%) | 3 (3.9%) | 2.42 (1.07) |
| **During MDRs** | | | | | | | |
| Q11 | Established EMR | 0 (0) | 1 (1.3%) | 17 (22.4%) | 49 (64.5%) | 9 (11.8%) | 3.87 (0.62) |
| | *i*-Dashboard | 0 (0) | 1 (1.3%) | 9 (11.8%) | 50 (65.8%) | 16 (21.1%) | 4.07 (0.62) |
| Q12 | Established EMR | 0 (0) | 4 (5.3%) | 21 (27.6%) | 44 (57.9%) | 7 (9.2%) | 3.71 (0.71) |
| | *i*-Dashboard | 0 (0) | 0 (0) | 9 (11.8%) | 47 (61.8%) | 20 (26.3%) | 4.15 (0.60) |
| Q13 | Established EMR | 1 (1.3%) | 6 (7.9%) | 21 (27.6%) | 40 (52.6%) | 8 (10.5%) | 3.63 (0.83) |

| | | | | | | | |
|---|---|---|---|---|---|---|---|
| | *i*-Dashboard | 0 (0) | 0 (0) | 7 (9.2%) | 46 (60.5%) | 23 (30.2%) | 4.21 (0.60) |
| Q14 | Established EMR | 0 (0) | 4 (5.3%) | 22 (28.9%) | 39 (51.3%) | 11 (14.5%) | 3.75 (0.77) |
| | *i*-Dashboard | 0 (0) | 0 (0) | 9 (11.8%) | 39 (51.3%) | 28 (36.8%) | 4.25 (0.66) |
| Q15 | Established EMR | 0 (0) | 3 (3.9%) | 23 (30.3%) | 40 (52.6%) | 10 (13.2%) | 3.75 (0.73) |
| | *i*-Dashboard | 0 (0) | 0 (0) | 8 (10.5%) | 39 (51.3%) | 29 (38.2%) | 4.28 (0.65) |

MDR, multidisciplinary round; EMR, electronic health record.

**Table S3.** Responses to Questionnaire 2 (n=76).

| Question | Response, n (%) | | | | | Points, mean (SD) |
|---|---|---|---|---|---|---|
| | Strongly disagree | Disagree | Neutral | Agree | Strongly agree | |
| Q1. *i*-Dashboard is my primary tool to gather data on patients. | 1 (1.3%) | 4 (5.3%) | 27 (35.5%) | 35 (46.1%) | 9 (11.8%) | 3.62 (0.82) |
| Q2. *i*-Dashboard is a tool that I am willing to use continuously. | 0 (0) | 3 (3.9%) | 14 (18.4%) | 40 (52.6%) | 19 (25.0%) | 3.99 (0.77) |
| Q3. *i*-Dashboard reduces time to recognize patient worsening and improving. | 0 (0) | 2 (2.6%) | 22 (28.9%) | 31 (40.8%) | 21 (27.6%) | 3.93 (0.82) |
| Q4. *i*-Dashboard increases the accuracy during data collection. | 0 (0) | 2 (2.6%) | 15 (19.7%) | 43 (56.6%) | 16 (21.1%) | 3.96 (0.72) |
| Q5. *i*-Dashboard reduces my workload. | 0 (0) | 3 (3.9%) | 26 (34.2%) | 28 (36.8%) | 19 (25.0%) | 3.83 (0.85) |
| Q6. *i*-Dashboard guides me in developing plans of care. | 0 (0) | 1 (1.3%) | 20 (26.3%) | 42 (55.3%) | 13 (17.1%) | 3.88 (0.69) |
| Q7. *i*-Dashboard makes me more effective as a team member. | 0 (0) | 0 (0) | 18 (23.7%) | 38 (50.0%) | 20 (26.3%) | 4.03 (0.71) |
| Q8. *i*-Dashboard decreases the complexity of my job. | 0 (0) | 6 (7.9%) | 21 (27.6%) | 34 (44.7%) | 15 (19.7%) | 3.76 (0.86) |
